# Impact of Buriti Oil Incorporation on the Structural and Functional Properties of Polymeric Films Based on Pectin and Sunflower Seed Protein

**DOI:** 10.3390/foods15213779

**Published:** 2026-10-22

**Authors:** Bárbara L. S. Freitas, Noemi P. Almeida, Leandro S. Oliveira, Adriana S. Franca

**Affiliations:** 1 Programa de Pós-Graduação em Ciência de Alimentos (PPGCA), Universidade Federal de Minas Gerais, Av. Antônio Carlos, 6627, Belo Horizonte 31270-901, MG, Brazil; barbaraluiza.s.f@gmail.com (B.L.S.F.); noemipalmeida@gmail.com (N.P.A.); leandro@demec.ufmg.br (L.S.O.); 2 Departamento de Engenharia Mecânica (DEMEC), Universidade Federal de Minas Gerais, Av. Antônio Carlos, 6627, Belo Horizonte 31270-901, MG, Brazil

**Keywords:** biopolymer, polysaccharide, *Mauritia flexuosa* L. f., byproduct, hydrophobicity, stiffness, durability, bio plasticizer

## Abstract

The demand for sustainable packaging has driven research into biopolymers as an alternative to conventional plastics. This study developed and characterized biodegradable films based on citrus pectin and sunflower seed protein (SSP), both in isolation and in blends, and investigated the use of buriti oil as a partial or total substitute for the plasticizer glycerol. The films were developed using a casting method and subjected to physicochemical, structural, optical, barrier, mechanical, and biodegradability analyses. The results indicated that the incorporation of the oil reduced moisture content and water solubility, especially in protein-rich formulations. Regarding barrier properties, the buriti oil emulsion maintained similar water vapor and oxygen resistance across most films, with improvements limited to pectin-based formulations for water vapor and to blended formulations for oxygen barrier. However, the complete replacement of glycerol compromised the mechanical properties, resulting in more brittle and less flexible materials, suggesting that the oil emulsion on its own does not act as an efficient plasticizer. The presence of the oil also controlled the rate of biodegradation, prolonging the decomposition time; however, it did not exceed 30 days. It was concluded that buriti oil is a promising functional additive for enhancing MC, WS and thermal stability; nonetheless, its application requires optimization, such as the use of plasticizer blends, to balance the structural stability and flexibility of the films.

## 1. Introduction

Packaging performs vital functions in protecting food from external contamination and physical damage. By ensuring product quality and safety, it extends shelf life and is essential for mitigating losses and waste [1,2]. However, the prevalence of petrochemical-based plastics in this sector has created an unsustainable environmental burden. The persistence of these non-biodegradable materials in the environment, coupled with the difficulty of recycling them and the potential release of toxic components, has heightened the urgency for renewable alternatives [3].

In line with the principles of circular economy, which advocate for the optimization of resources and the minimization of waste, the transition to biodegradable packaging has strongly driven the valorization of agri-food byproducts [4]. Far from being mere waste, these residues are rich and abundant sources of biopolymers, such as polysaccharides and proteins [5], emerging as strategic matrices for conversion into new materials with high added value and low environmental impact.

A notable example of these biopolymers extracted from waste is pectin, a complex structural heteropolysaccharide that can be obtained from fruit processing waste, such as citrus peels and apple pomace [6]. Similarly, sunflower seed protein (SSP), a valuable byproduct of oil extraction, is often underutilized [7]. Both classes of biopolymers (polysaccharides such as pectin and plant proteins such as SSP) have been extensively studied to produce biodegradable films due to their abundance and environmentally favorable characteristics [1]. However, films based purely on these biopolymers have severe technical limitations, tending to be excessively rigid and brittle, highly permeable, and highly hydrophilic [2,5,6]. The development of polysaccharide and protein blends has served as a strategy to improve these conditions, allowing the synergy between the polymers to expand the applications of these materials in the packaging sector [3].

Furthermore, an even more effective strategy for overcoming these limitations has been the addition of plasticizers designed to reduce intermolecular interactions between polymer chains, resulting in more flexible and processable films, which are desirable characteristics for packaging materials [5,6]. Glycerol is one of the most used plasticizers in biodegradable films due to its effectiveness and low cost. However, its high hygroscopicity can compromise the stability of the films in humid environments, negatively affect their water barrier properties, and lead to its migration from the polymer matrix over time, altering the film’s properties. These limitations have driven the exploration of alternative plasticizers, preferably from renewable sources, that can not only provide the desired plasticity but also add functionalities.

To overcome the limitations of biopolymers and the inefficiency of conventional plasticizers, many studies have turned to the addition of reinforcing agents, biopolymers, and natural agents, such as essential oils and vegetable oils [8]. Vegetable oils, composed primarily of triglycerides formed by glycerol and three fatty acid chains of varying lengths and degrees of saturation [9], stand out for their dual functionality, acting as barrier agents and exerting a plasticizing effect. It is suggested that their more flexible fatty acid chains can disperse and intercalate between the polymer chains of the matrix, increasing intermolecular spacing, segmental mobility, and, consequently, the flexibility and processability of the film [2,9]. The incorporation of these lipids into hydrophilic polymer matrices, such as those composed of polysaccharides and proteins, aims to reduce their sensitivity to water and improve water vapor barrier properties [2]. By imparting greater hydrophobicity to the surface and internal structure of the material, oils minimize moisture absorption from the environment, a critical factor for the preservation of food products [10].

Recent literature corroborates the success of this strategy: polysaccharide films, particularly those based on pectin, have demonstrated excellent performance when incorporated with lemon, orange, and oregano essential oils [11,12,13]. Similarly, protein matrices have benefited significantly from lipid plasticization, as evidenced by zein films plasticized with castor oil [14], whey protein films with marjoram oil [15], and sunflower protein-based films containing eugenol-loaded emulsions [16].

The vast Brazilian biodiversity includes buriti oil (*Mauritia flexuosa* L. f.), which stands out as a resource of great ecological and sociocultural significance [17]. Its lipid composition consists predominantly of triglycerides and is notably rich in unsaturated fatty acids. Oleic acid (C18:1) is the major component, followed by palmitic acid (C16:0) and smaller fractions of stearic and linolenic acids [18,19]. However, the key technological distinction of this oil lies in its content of bioactive compounds, particularly tocopherols and carotenoids, such as β-carotene, which confer promising functional properties [18]. From the perspective of circular economy, it is important to note that this oil is extracted primarily through mechanical pressing. This process generates a residual byproduct that still retains a significant lipid content, making it feasible to use it as an affordable, high-value-added raw material for the development of new materials. Thus, it has emerged as a promising candidate for imparting hydrophobic and functional properties to films [2,9,18].

Based on these characteristics, buriti oil (BO) has been explored as an alternative plasticizer and additive in various polymer matrices. Durães et al. [20] demonstrated that the incorporation of the oil plasticized polystyrene (PS) and polymethyl methacrylate (PMMA), conferring greater thermal stability due to its bioactive compounds. Promising results were also obtained by Bispo-Jr et al. [18] in PVDF composite membranes, where the addition of BO promoted plasticization of the material and a significant improvement in thermal stability, hindering polymer degradation. In the field of biopolymers, Anjos et al. [21] reported that the addition of BO was crucial for reducing the water vapor permeability of gelatin-based protein matrices. Furthermore, the oil imparted active properties to the packaging, such as antioxidant and bacteriostatic activity, significantly inhibiting the growth of pathogens during storage. In a previous study, Freitas et al. [22] observed that the addition of buriti oil (BO) to carbohydrate-based films (starch, galactomannan, or both) inhibited retrogradation (in starch films and composite films), while also improving water resistance and barrier properties (in galactomannan films). The addition of BO resulted in distinct interactions among the components and, consequently, in films with different properties. Given that the use of polysaccharide and protein blends has been a successful strategy for improving polymeric film properties, and that studies using BO have been restricted only to polymeric films based on either gelatine or carbohydrates (starch and galactomannan), there is still a gap in this research area that could lead to a better understanding of the interaction between film components in order to produce biodegradable and renewable polymeric films. Moreover, given that buriti oil can impart a plasticizer effect in the polymeric matrix, we herein innovate in utilizing such oil in polymeric matrices that have not yet been studied for such endeavor, such as pure pectin and blends of pectin and protein.

Therefore, the goal of this study was to thoroughly investigate the effect of adding buriti oil as an alternative plasticizer to biopolymer films based on citrus pectin and sunflower seed protein (SSP) matrices, both individually and in blends. By filling this gap, the study contributes to advances in the development of biodegradable packaging, combining technological improvements with the valorization of a strategic resource from the Brazilian Cerrado region.

## 2. Materials and Methods

### 2.1. Materials

The reagents used to prepare the films included citrus pectin with 70 to 85% esterification (Dinâmica Química Contemporânea, Indaiatuba, SP, Brazil); sunflower seed protein with low chlorogenic acid content (PISG–SUNPROTEIN, 80% crude protein), donated by “Bio Technologies” LLC (Moscow, Russia); glycerol (Synth, Diadema, SP, Brazil); pure powdered gum arabic density 1.35 (Êxodo Científica, Sumaré, SP, Brazil); and buriti oil (batch MFR 24001), donated by Cooperativa Grande Sertão (Montes Claros, MG, Brazil). The other reagents used for the film characterization analyses were of analytical grade.

### 2.2. Film Preparation

The films were produced by the casting method following the methodologies of Alves et al. [23] and Siqueira et al. [24], with some modifications. Pectin was dispersed in water under constant shaking for 1 h and 30 min at room temperature. The solution was then heated to 70 °C and maintained at that temperature for an additional 30 min while stirring. After heating had ceased, glycerol and the buriti oil emulsion were added as soon as the temperature dropped below 50 °C. The dispersion was placed in an ultrasonic bath (Unique, Indaiatuba, Brazil, model USC-165, In) (25 kHz, 150 W) for 30 min, until all bubbles were removed.

For the sunflower seed protein films, after dispersing the polymer in water, the pH was adjusted to 11 with 2 M sodium hydroxide solution and the solution was maintained under constant agitation for 30 min at room temperature. Subsequently, glycerol and the buriti oil emulsion were added to the mixture and homogenized for 5 min. Finally, the film-forming solution was sonicated for 15 min in an ultrasonic bath (Unique, model USC-165A) (25 kHz, 150 W), followed by 2 min of probe sonication (Ultronique, Indaiatuba, Brazil, Disruptor model, QR350) at 85 kHz, to ensure the complete removal of any bubbles present in the mixture.

To prepare the buriti oil-in-water emulsion, gum arabic was used as the emulsifying agent, in proportion to the amount of oil used. Both were mixed using a probe sonicator (model QR500, Ultronique, Eco-Sonics, Indaiatuba, SP, Brazil) operating at 20 kHz and 425 W for 3 min. For the films containing pectin and protein in their composition, each solution was prepared independently, following the gelling process specific to each polymer, and the solutions were mixed just prior to the addition of the buriti oil emulsion and glycerol.

Approximately 138 g of the film-forming solutions were poured into silicone molds (17.5 cm^2^) and dried in a convection oven with air circulation (Nova Ética, São Paulo, Brazil model 420-1DE) at 30 °C for 24 h. The films were carefully removed from the molds and, before being analyzed, the samples were stored in a controlled environment at 20 °C and a relative humidity of approximately 40% for a maximum of 3 months until the characterization analyses were completed. An overview of the films’ composition is provided in Table 1.

It should be noted that the addition of gum arabic to F2 and F3 was necessary solely for the emulsification of buriti oil. Previous studies [22] indicate that gum arabic acts as a neutral carrier without drastically altering the mechanical properties in similar matrices. Although the structural changes in the composite films have been mainly associated with the lipid phase, the presence of the emulsifier is recognized as an inherent variable in the system.

### 2.3. Film Characterization

#### 2.3.1. Chromatic Properties and Thickness

The colorimetric analysis in this study was conducted using a tristimulus colorimeter (Colorflex, Hunter Associates Laboratory, Reston, VA, USA) by measuring the L^*^, a^*^, and b^*^ parameters under standard D65 illumination and a normal colorimetric viewing angle of 10° [23,25]. Parameters L^*^, a^*^ and b^*^ (CIELAB color space) represent lightness (L^*^) and the coordinates in the green-red (a^*^) and blue-yellow (b^*^) axis. Based on the results, the chroma (c) and hue angle (h) properties were calculated using the following equations:

(1) $$c^{*}=\sqrt{a^{*2}+b^{*2}}$$

(2) $$h^{*}=180+\sqrt{\tan\frac{b^{*}}{a^{*}}}\;\;\;\;a^{*}\;<\;0\;\mathrm{and}\;b^{*}\;>\;0$$

(3) $$h^{*}=\sqrt{\tan\frac{b^{*}}{a^{*}}}\;\;\;\;\;\;a^{*}\;\mathrm{and}\;b^{*}\;>\;0$$

To calculate opacity, the absorbance of the films was measured using a UV-vis spectrophotometer (Spectrum, Stamford, CT, USA, model SP-2000UV) at a wavelength of 600 nm in quartz cuvettes. The obtained values were used to calculate the results using the following equation:

(4) $$\mathrm{Opacity}=\;\frac{A_{600}}{x}$$

where A_600_ is the absorbance of the sample at 600 nm, and *x* is the film thickness [23].

The thicknesses of the samples were measured using an external micrometer (103-137, Mitutoyo Corporation, Kawasaki, Japan), with a precision of 0.001 mm, at 24 random points distributed across the same film. The thickness values were used to evaluate the water vapor permeability and mechanical properties of the films.

#### 2.3.2. Scanning Electron Microscopy (SEM)

The surface and cross-sectional morphology of the films was evaluated using an FEI Quanta 200 FEG scanning electron microscope (FEI Company, Hillsboro, OR, USA). The samples were mounted on metal holders using double-sided carbon adhesive tape and metallized with a gold/palladium (Au/Pd) layer approximately 5 nm thick.

#### 2.3.3. Infrared Spectroscopy (FTIR)

Functional characterization of the films was performed using a Thermo Scientific Nicolet 6700 FTIR spectrophotometer (Thermo Fisher Scientific, Waltham, MA, USA) coupled to an attenuated total reflectance (ATR) accessory with a ZnSe crystal, with detection via DLATGS (deuterated triglycine sulfate doped with L-alanine). The scan range was 4000–600 cm^−1^ with a resolution of 4 cm^−1^ and 16 scans.

#### 2.3.4. Mechanical Properties

Tensile strength (TS), modulus of elasticity (EM), and elongation at break (EB) were determined using a texture analyzer (TA.XT Plus, Stable Micro Systems, Godalming, UK), equipped with a 5 kg load cell, with an initial distance between the grips of 50 mm and a strain rate of 0.8 mm/min. The tests were conducted on 10 mm × 100 mm samples, with at least five replicates based on films from different production batches.

#### 2.3.5. Thermogravimetric Analysis (TGA)

The thermal stability and degradation of the films were evaluated by thermogravimetric analysis (TGA), performed using a TA Instruments Thermal Analyzer (Model SDT Q600, New Castle, DE, USA). Approximately 10 mg of each sample were placed in aluminum crucibles and subjected to a temperature range of 25 to 600 °C at a heating rate of 10 °C min^−1^, under a nitrogen atmosphere of 50 mL min^−1^ [23].

#### 2.3.6. Moisture Content (MC) and Water Solubility (WS)

The moisture content of each film was determined using the standard gravimetric method, in which the final and initial weights of the samples (2 cm × 2 cm), dried at 105 °C for 24 h, were compared. For water solubility (WS) analyses, the method of Gontard et al. [26] was used with modifications. Film samples measuring 2 × 2 cm were dried at 105 °C for 24 h and weighed; they were then immersed in 30 mL of distilled water and stirred at 150 rpm for 24 h. The samples were filtered, dried, and weighed again to quantify WS using the following equation:

(5) $$\mathrm{WS}=\;\frac{\mathrm{initial}\;\mathrm{weight}-\mathrm{final}\;\mathrm{weight}}{\mathrm{initial}\;\mathrm{weight}}\;\times 100\%$$

#### 2.3.7. Barrier Properties

Barrier property analyses were performed following the methodology of Alves et al. [23], Yan et al. [27] and Sarker, Matak, and Jaczynski [28]. To determine water vapor permeability (WVP) and water vapor transmission rate (WVTR), samples of each film were cut and attached to the ends of glass tubes containing pre-dried silica (~1/3 of the tube’s volume) and placed in a controlled environment (20 ± 2 °C and 75% relative humidity). The weight of the tubes was monitored daily for 7 days to calculate the WVP and WVTR values using the following equations:

(6) $$\mathrm{WVP}=\;\frac{w}{t}\times \;\frac{\delta }{\left(\Delta P\times A\right)}$$

(7) $$\mathrm{WVTR}=\frac{w}{t}\times \frac{1}{A}$$

where $\frac{w}{t}$ is the slope of the weight gain versus time curve (g/day); *A* is the permeation area of the sample (m^2^); *δ* is the sample thickness (mm); and Δ*P* is the variation in water vapor saturation pressure across the film at the experimental temperature (1.75425 kPa).

To calculate oxygen permeability (OP), 3 g of a deoxidizing reagent (iron powder, activated carbon, and sodium chloride, in a ratio of 0.5:1:1.5 (*w*/*w*/*w*)) were added to 15 mL glass vials and covered with the films. The vials were kept in a desiccator containing a saturated barium chloride solution (90% RH) for 48 h at 25 °C. First, the Oxygen Transmission Rate (OTR) was calculated from the mass gain of the vials using the following equation:

(8) $$\mathrm{OTR}=\;\frac{\Delta w}{t\times A}$$

where Δw is the mass difference, *t* is the equilibrium time in seconds, and *A* is the permeation area of the sample (m^2^).

Subsequently, the true Oxygen Permeability (OP) was determined by normalizing OTR according to Equation (9):

(9) $$\mathrm{OP}=\frac{\mathrm{OTR}\times L}{\Delta P}$$

where L is the average film thickness (mm) and ΔP is the partial pressure gradient of oxygen across the film (kPa). To ensure strict thermodynamic accuracy, the ΔP (20.68 kPa) was calculated considering Dalton’s Law of Partial Pressures, by subtracting the partial vapor pressure of water generated at 90% RH and 25 °C from the standard atmospheric pressure before applying the 21% atmospheric oxygen fraction.

#### 2.3.8. Biodegradability

The biodegradability analysis of the films was based on a qualitative photograph-based test to determine the number of days it takes for the bioplastic to disintegrate in controlled soil conditions. This test was conducted following the method described by Alves et al. [23], with modifications. Film samples (2 × 2 cm) placed over plastic meshes were buried in plastic containers filled with agricultural soil and peat-type soil (All Garden, Holambra, Brazil) and kept at room temperature. For the analysis, the soil was regularly sprayed with distilled water, and the samples were removed and analyzed daily until they were completely disintegrated, which, in this context, means that they are no longer visible on the mesh.

#### 2.3.9. Statistical Analysis

All analyses were performed in triplicate (three measurements taken from randomly selected pieces from same film batch). Calculations of the mean, standard deviation, analysis of variance (ANOVA), and Tukey’s test for comparing means (*p* < 0.05) were performed using Minitab Statistical Software, version 21, State College, PA, USA.

## 3. Results and Discussion

### 3.1. Chromatic Properties and Thickness

**Figure 1:**
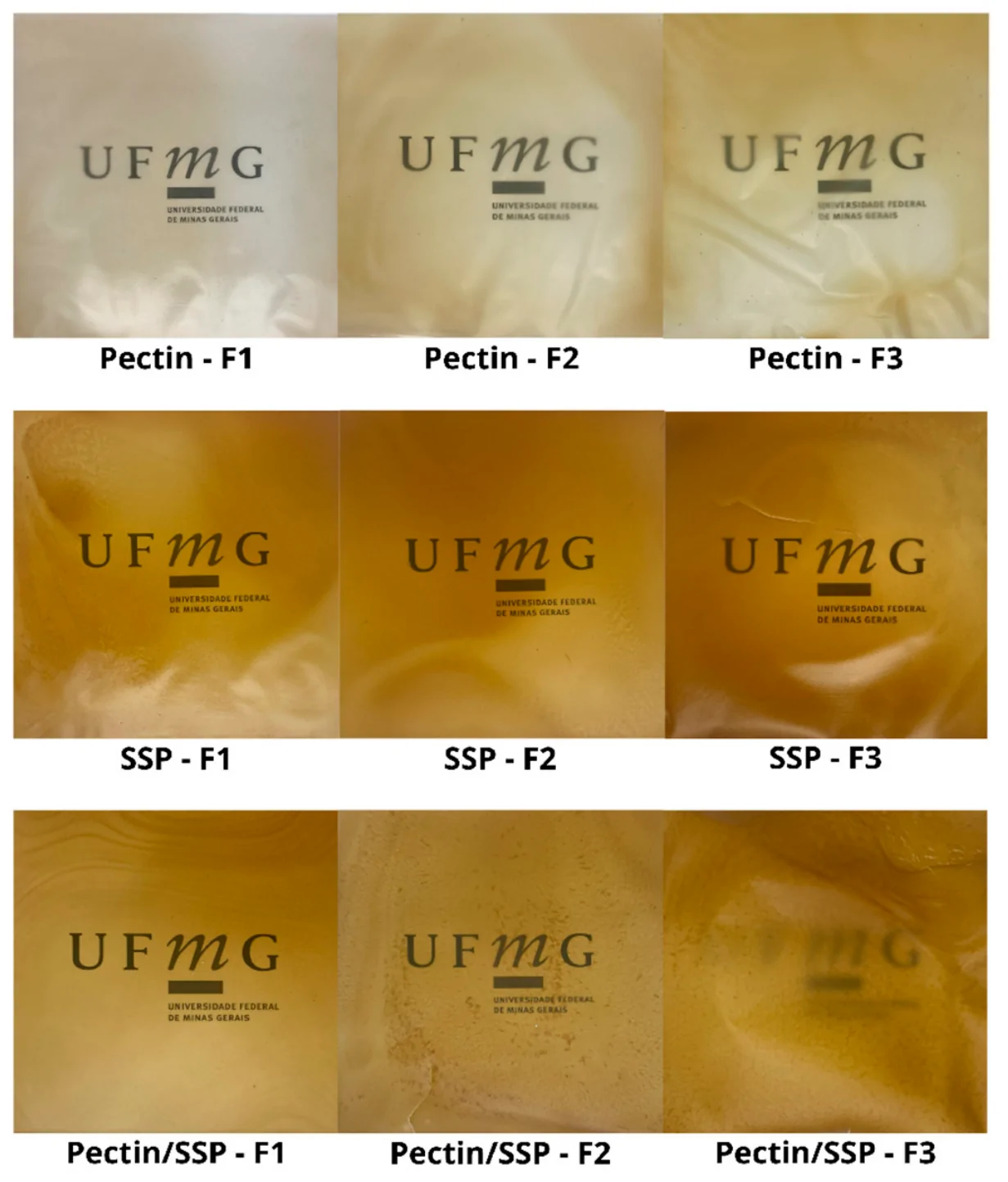
Photographs of the prepared films. (F1) control films; (F2) films with partial replacement of glycerol by buriti oil emulsion; (F3) films with total replacement of glycerol by buriti oil emulsion.

The optical properties of the films varied significantly depending on the polymer matrix and on the amount of added buriti oil. Photographs of the prepared films are shown in Figure 1 and color parameters (L^*^, c^*^, h^*^ and opacity) are displayed in Table 2.

Regarding lightness (L^*^), which can range from 0 (black) to 100 (white), the pure pectin films exhibited the highest values, with a significant increase following the addition of buriti oil emulsion. For SSP films, the partial substitution of glycerol by BO emulsion (F2) led to a significant increase in luminosity value, followed by a small decrease after total glycerol substitution (F3). In contrast, no significant changes in L^*^ values were observed in the blended films. This behavior is consistent with the visual coloration observed in the images, where the protein films containing oil seem to be darker and more opaque.

Color saturation (c^*^) increased in all F3 formulations with the incorporation of the emulsion when compared with F1, indicating that buriti oil emulsion acts as a color-intensifying agent, except for SSP, which had no statistical difference [29]. The h^*^ parameter (hue angle), which represents the perceived color type (hue), varied little among the treatments, remaining in the range of 70° to 103°, indicating that the dominant color remained in the yellow spectrum (~90°). This can be related to the rich carotenoid content of buriti oil [19,22].

The changes in color parameters are related to how the oil droplets behave with other molecules in the matrix; therefore, it will not always respond in the same way. For example, Gomes et al. [16] observed that the addition of sunflower oil to an SSP film resulted in a lighter, more intense, and yellower film. When comparing the current results with a previous study using starch and galactomannan matrices [22], the reproducibility of this behavior becomes evident: the incorporation of the oil increases saturation and shifts the color toward the yellow spectrum, demonstrating that its natural pigmentation prevails over the inherent visual characteristics of different polymeric matrices. This strong coloring capacity of buriti oil has also been reported when applied to emulsions [30].

The opacity of the films was also altered by the addition of buriti oil emulsion. In the pectin films, there was a significant increase in opacity upon complete replacement of glycerol with buriti oil emulsion, a finding that may be related to an increase in light scattering due to the higher incorporation of lipid compounds [31]. In protein films, however, a reduction in opacity was observed compared to the control. In the pectin/SSP blends, instrumental opacity remained statistically stable across the formulations. However, visual observation revealed an increase in turbidity, especially in formulation F3, likely due to the light scattering effect of the lipid phase dispersed in the matrix, suggesting a matrix with intermediate visual behavior.

Regarding thickness, it was observed that replacing glycerol with buriti oil emulsion led to significant changes, especially in formulations containing proteins. This increase can be attributed to the hydrophobic interaction between the protein constituents and the oil, which can alter the conformation of the polymer network and result in thicker films [32].

These results demonstrate that the incorporation of buriti oil emulsion directly influences the visual characteristics of the films, resulting in more intense coloration, especially in formulations with higher protein content. Modulation of color and opacity is desirable, depending on the material’s end use, either as translucent packaging or packaging with a light-barrier function [33].

### 3.2. Scanning Electron Microscopy (SEM)

**Figure 2:**
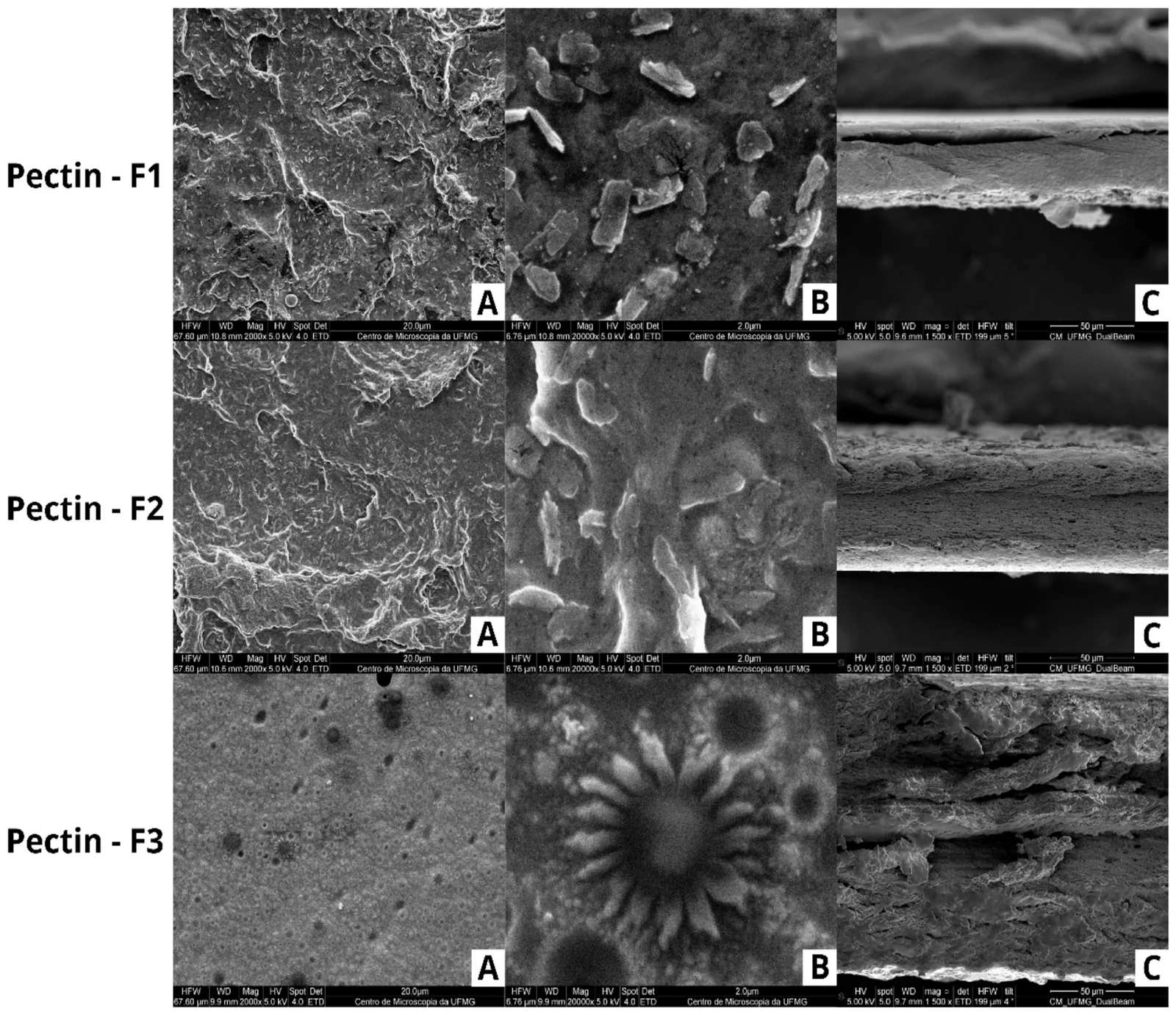
Microscopic images of pectin-based films using SEM at (**A**) 2000×, (**B**) 20,000× and (**C**) cross-section 1500×. (F1) Control films; (F2) films with partial replacement of glycerol with buriti oil emulsion; (F3) films with total replacement of glycerol with buriti oil emulsion.

**Figure 3:**
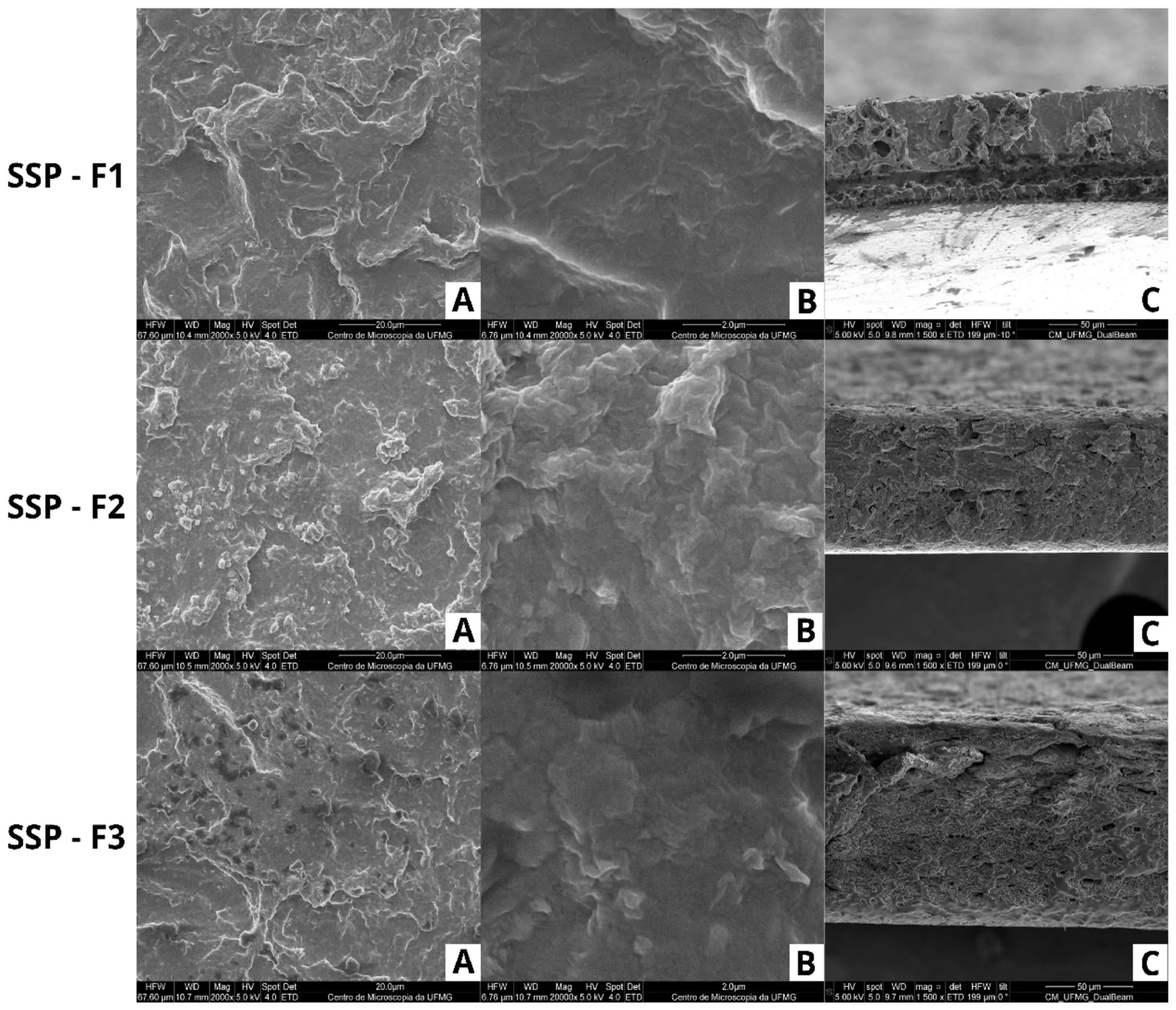
Microscopic images of SSP-based films using SEM at (**A**) 2000×, (**B**) 20,000× and (**C**) cross-section 1500×. (F1) Control films; (F2) films with partial replacement of glycerol with buriti oil emulsion; (F3) films with total replacement of glycerol with buriti oil emulsion.

**Figure 4:**
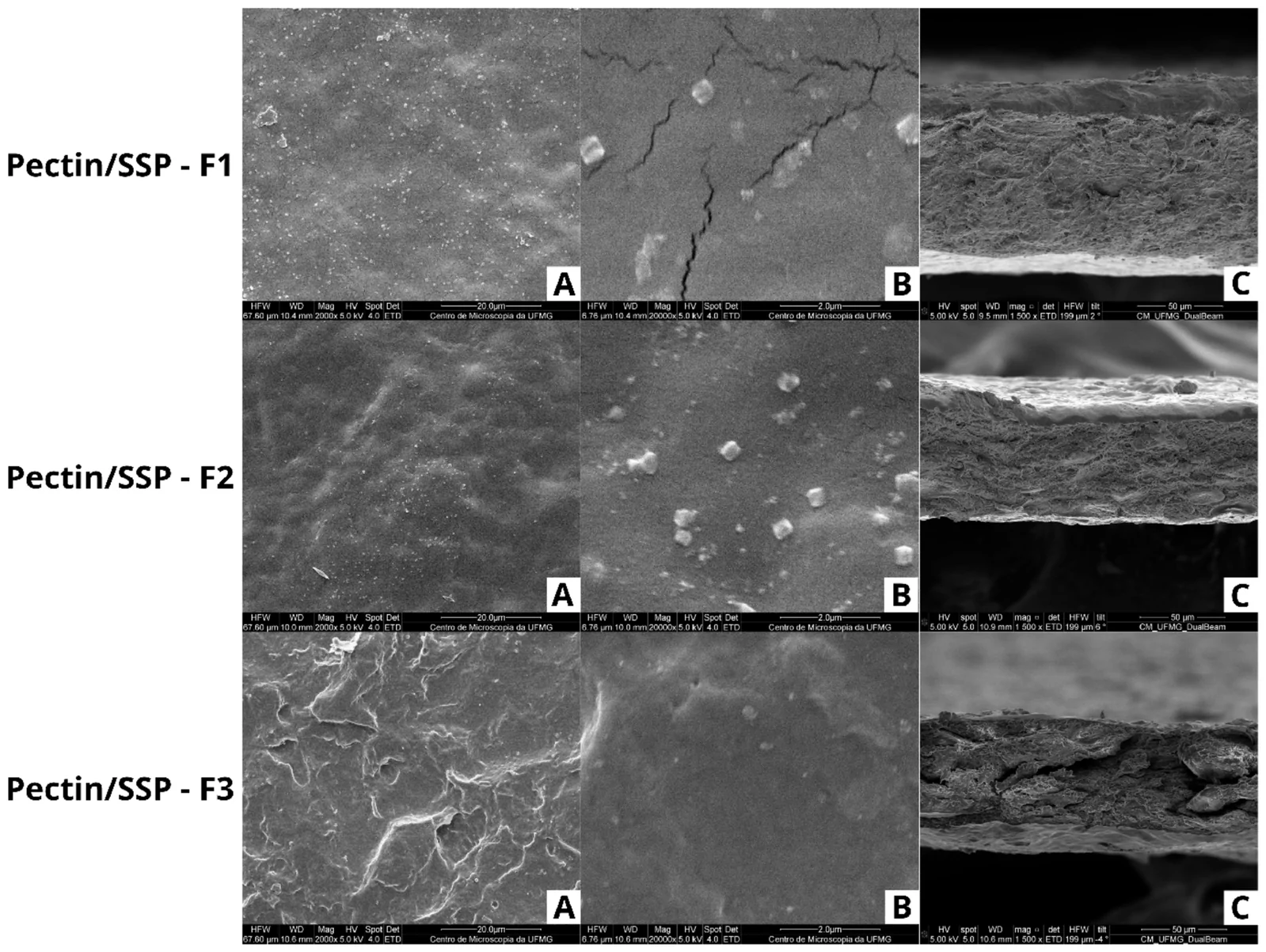
Microscopic images of blended films using SEM at (**A**) 2000×, (**B**) 20,000× and (**C**) cross-section 1500×. (F1) Control films; (F2) films with partial replacement of glycerol with buriti oil emulsion; (F3) films with total replacement of glycerol with buriti oil emulsion.

The evaluation of morphology by scanning electron microscopy (SEM), shown in Figure 2, Figure 3 and Figure 4, provided insight into the impact of replacing glycerol with buriti oil emulsion in the different polymer matrices studied. The micrographs revealed that compatibility and structural organization depend heavily on the nature of the cross-linking biopolymer. As Durães et al. [20] reported, the addition of BO can lead to different behaviors, as it is correlated to the way the oil coagulates within the film structure as the solvent evaporates.

The F1 and F2 films composed exclusively of pectin, shown in Figure 2, exhibited a significantly more cohesive, dense, and compact matrix compared to the films containing protein. The control formulation (F1) exhibited a continuous, smooth cross-sectional structure free of visible pores, a characteristic intrinsic to the packing of the chains of this polysaccharide when properly plasticized with glycerol [34]. This integrity was satisfactorily maintained in formulation F2. However, the complete replacement of the hydrophilic plasticizer with buriti oil emulsion (F3) caused drastic changes in the microstructure. A significant appearance of bubbles and micro-holes was observed, a phenomenon directly related to the drying process: the presence of a large amount of free oil may have caused the rapid evaporation of water and the volatilization of compounds to disrupt the polymer matrix [35]. This mechanical rupture is evident in the surface image F3(B), which exhibits an irregular, radial “rosette” or floral structure, that could be due to solvent escape pathways or severe droplet coalescence, and in the cross-section F3(C), where cracks can be seen. Such imperfections indicate that an excess of lipid vesicles could cause a disruption in the continuous polymeric network of the carbohydrate, creating points of structural failure [36].

The morphological behavior of films composed exclusively of SSP differed substantially from the others. In the cross-section of the control formulation F1(C), the formation of a thin, extremely dense, and cohesive layer was identified in the lower portion, sandwiched between a less structured and thicker layer in the upper portion. Unlike the matrices containing pectin, the addition of emulsified buriti oil (F2 and F3) did not result in significant microscopic changes and did not induce the formation of the characteristic cracks observed in the other matrices. This maintenance of structural stability suggests that the protein network is well-compatible with the lipid emulsion used, interacting in a way that preserves the matrix’s state without causing phase separation or the abrupt discontinuity that leads to excessive porosity [37].

For the composite films, the microstructural behavior proved to be distinct. Starting with the control formulation (F1), the matrix appeared noticeably more irregular, with the presence of numerous cracks distributed throughout its length. This initial disorganization may result from heterogeneous packing and complex interactions between the protein chains and the polysaccharide during film drying [36]. Partial replacement with buriti oil (F2) did not cause any additional severe morphological differences compared to the control. However, in the case of total replacement (F3), the cross-section showed a noticeable increase in the occurrence of cracks. Nevertheless, it is essential to note that the level of cracks in the F3 blend films was lower compared to that of the pure pectin films with the same lipid loading. This suggests that the blended matrix can accommodate the hydrophobic phase slightly more efficiently, possibly due to noncovalent interactions between the proteins and the oil [37].

### 3.3. Infrared Spectroscopy (FTIR)

Before discussing the spectroscopic results, it is important to understand the structural characteristics of the biopolymers that make up the matrix. Commercially available citrus pectin with a high degree of methoxylation is a rigid linear polysaccharide whose main chain consists primarily of α-(1,4)-D-galacturonic acid units [5,38]. Due to its high degree of esterification, the molecule contains an abundant number of hydrophilic and amphiphilic groups, specifically hydroxyl groups (–OH) and alkyl ester groups (COOCH_3_), as well as a fraction of free carboxylic acids [5,6]. This structure makes it a reasonably polar macromolecule, highly dependent on water-mediated hydrogen-bond networks and hydrophilic plasticizers to maintain its mobility and interfacial stability [5,6]. In contrast, sunflower seed protein (SSP) is composed predominantly of the 11S globulin fraction (helianthin) [7,39]. SSP is rich in amino acids with charged side chains (such as the carboxylate groups of glutamic and aspartic acids, and the guanidinium cations of arginine) and regions formed by nonpolar amino acids [40]. This structural versatility, combined with the ability to transition between β-sheet and α-helix conformations, confers an amphiphilic character to the protein, allowing it to interact with both polar matrices and lipid components through rearrangements in its hydrophobic domains [41].

**Figure 5:**
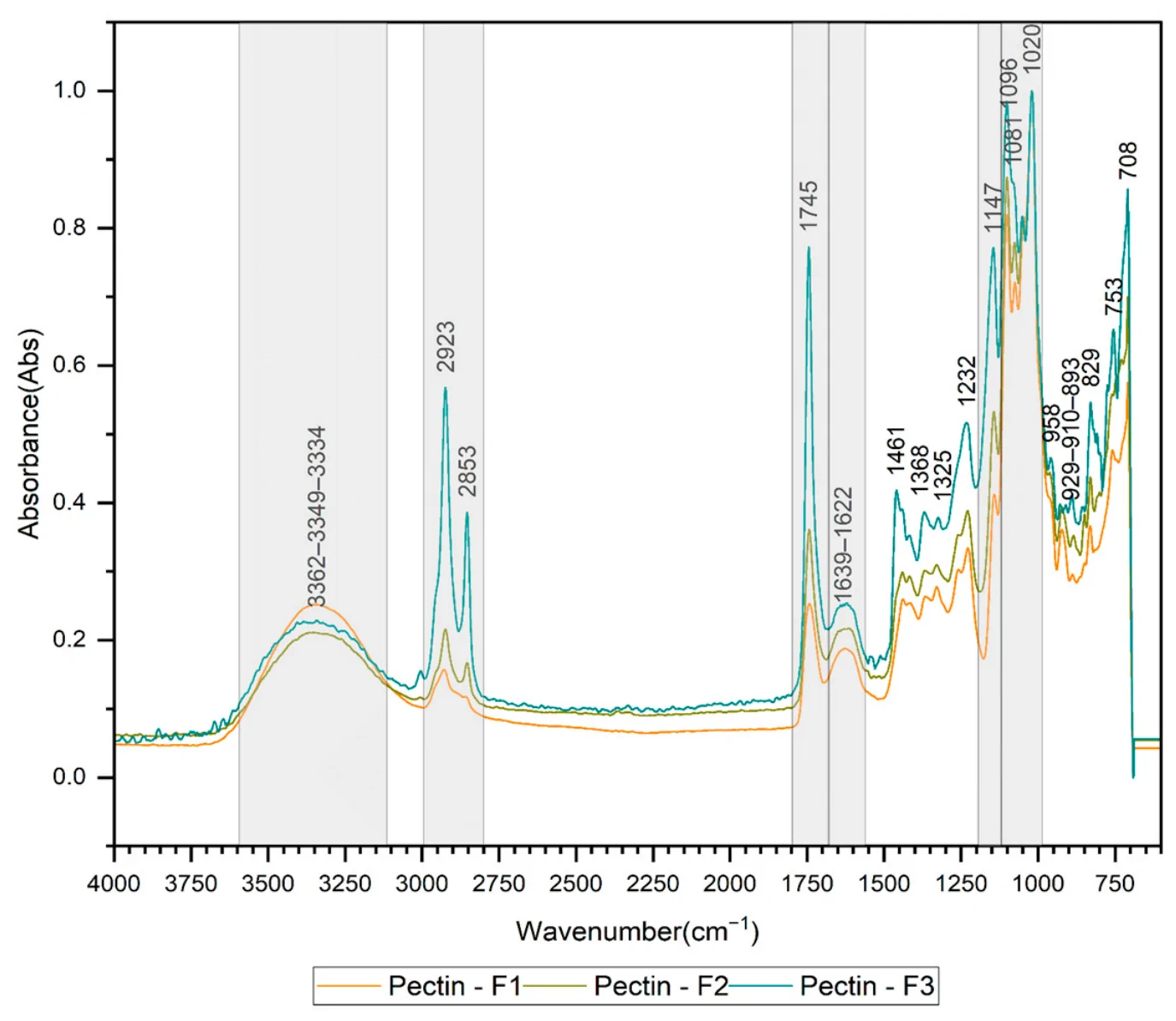
FTIR spectra for the pectin-based films. (F1) control films; (F2) films with partial replacement of glycerol with buriti oil emulsion; (F3) films with total replacement of glycerol with buriti oil emulsion. Gray shaded regions indicate the main spectral bands discussed in this section.

**Figure 6:**
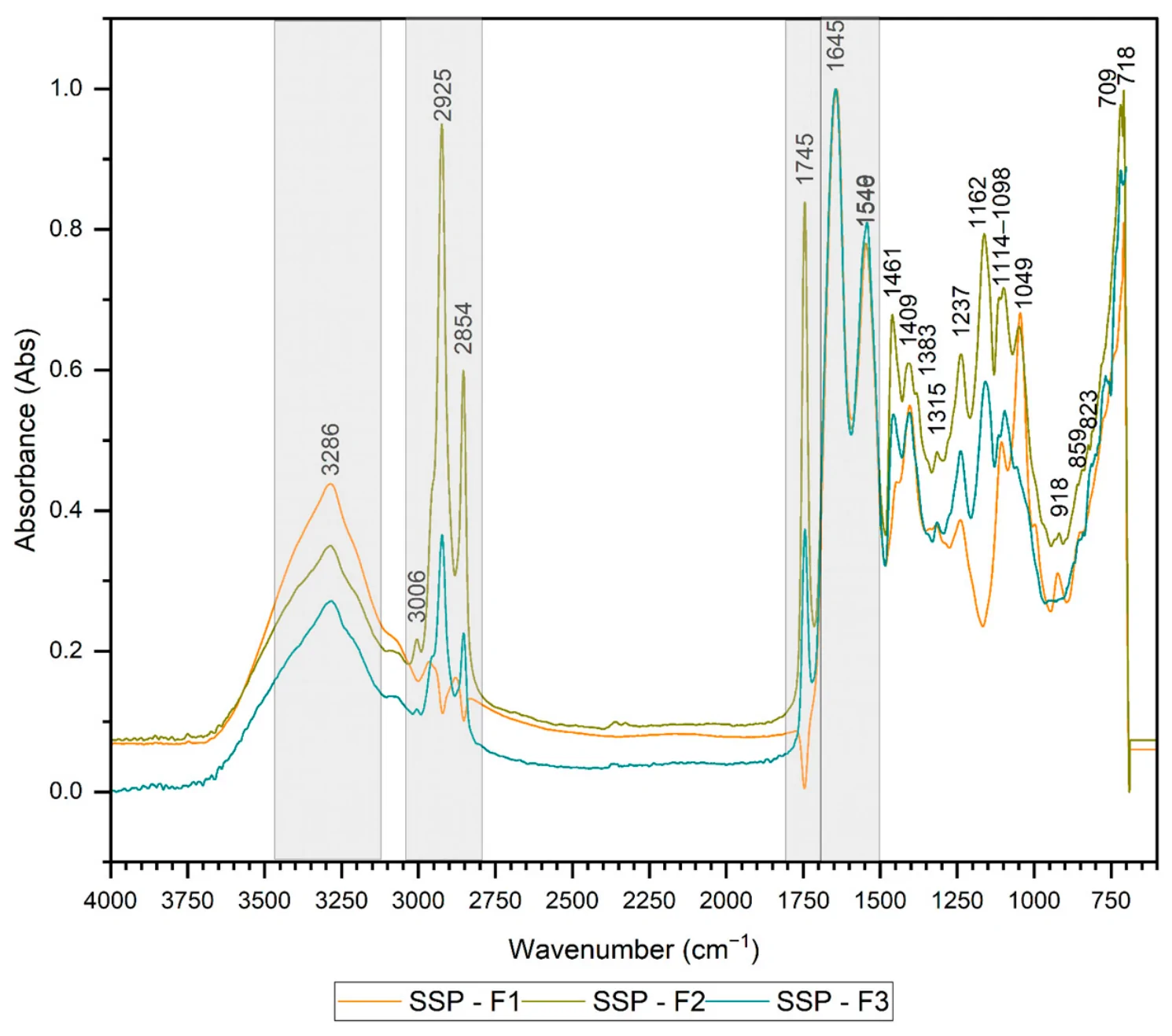
FTIR spectra for the SSP-based films. (F1) control films; (F2) films with partial replacement of glycerol with buriti oil emulsion; (F3) films with total replacement of glycerol with buriti oil emulsion. Gray shaded regions indicate the main spectral bands discussed in this section.

**Figure 7:**
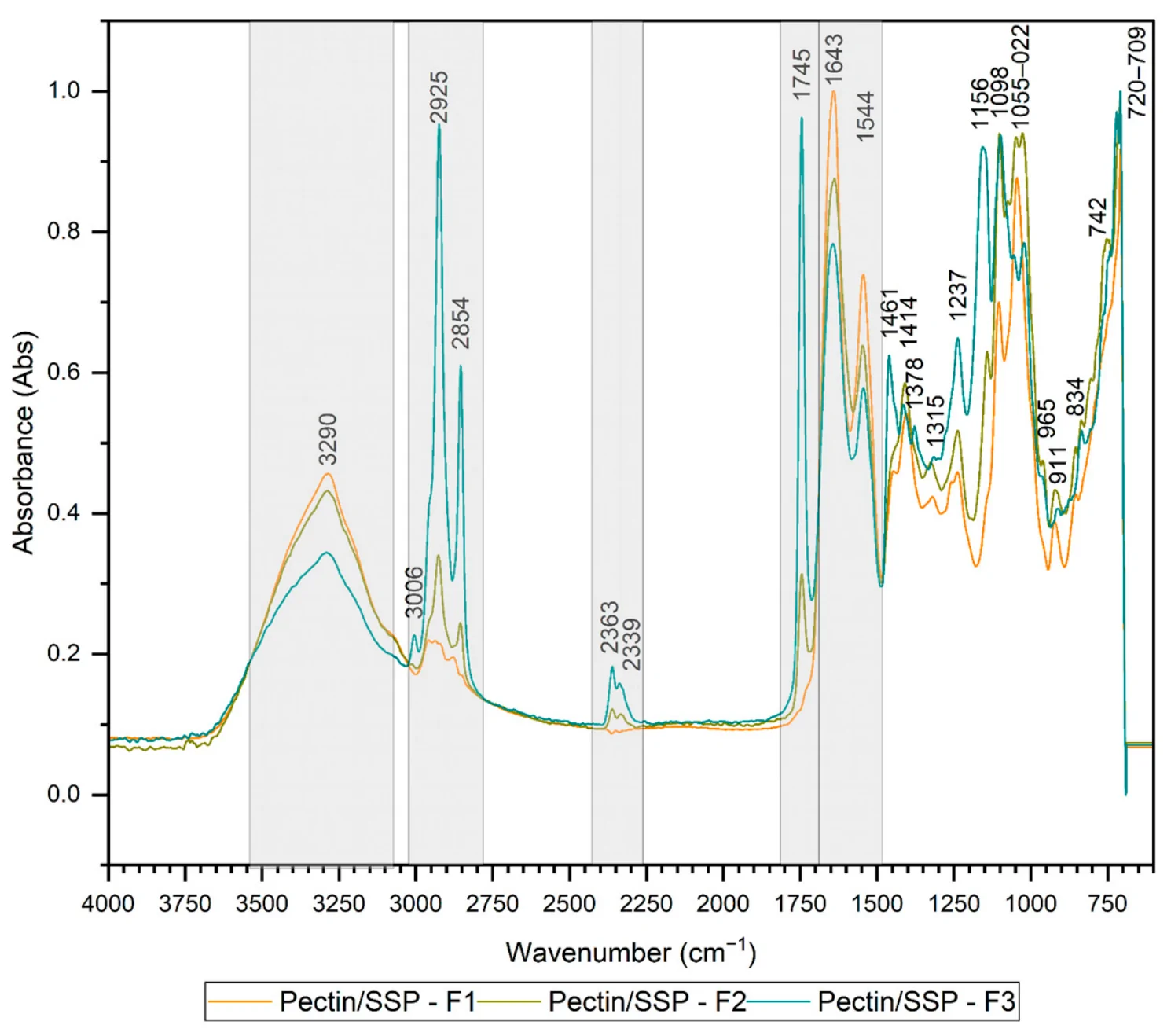
FTIR spectra for the blended films. (F1) control films; (F2) films with partial replacement of glycerol with buriti oil emulsion; (F3) films with total replacement of glycerol with buriti oil emulsion. Gray shaded regions indicate the main spectral bands discussed in this section.

An analysis of the obtained spectral data allowed for a detailed evaluation of the molecular interactions and structural changes resulting from the different polymeric and lipid compositions, as shown by the spectra detailed in Figure 5, Figure 6 and Figure 7. To refine the vibrational interpretation, particularly in the complex fingerprint region (ranging from 1400 to 700 cm^−1^), the second derivative technique was applied to the spectra. The use of the second derivative significantly improves sensitivity in identifying chemical changes, allowing for improved resolution of overlapping bands and the detection of specific interactions within the matrix [42].

In the pectin films, the F1 control sample exhibited a characteristic spectral profile. A broad O-H stretching band was observed, peaking at ~3362 cm^−1^, indicative of hydrogen bonds between the hydroxyl groups of pectin, glycerol, and water [43]. The C–H stretching peaks were of low intensity, located at ~2923 cm^−1^ and with a shoulder at ~2853 cm^−1^. A prominent band at ~1745 cm^−1^, slightly more intense, is attributed to the C=O stretching of the native alkyl ester groups in highly esterified pectin [43]. An overlapping band at ~1639–1622 cm^−1^, likely related to the deformation vibration of adsorbed water molecules and free carboxyl groups of pectin, was also observed [25]. Water exhibits a prominent H–O–H bending vibration at ~1639 cm^−1^, whereas ionized free carboxyl groups exhibit an asymmetric stretching vibration at ~1630 cm^−1^. The fingerprint region showed peaks at ~1147 cm^−1^, associated with the glycosidic bonds of pectin, and a set of intense bands between 1100 and 1000 cm^−1^, possibly associated with the stretching vibrations of C-O and C-C bonds in the rings of the pectin main chain [43,44].

With the partial replacement of glycerol with buriti oil emulsion in the F2 film, the O-H band (~3349 cm^−1^) was found to be slightly shifted and of lower intensity compared to F1. The shift in this band indicates that the addition of the emulsified lipid altered the original hydrogen bonds, forcing a restructuring of the polar environment of the pectin matrix. There was a considerable increase in the intensity of the C–H peak at ~2853 cm^−1^, confirming the presence of saturated aliphatic chains introduced by the lipid component [45]. Notably, the ester C=O peak at ~1745 cm^−1^ showed an increase in intensity compared to F1, suggesting that the contribution of the C=O stretching of the oil triglycerides was added to that of the pectin esters [45]. The band at ~1639–1622 cm^−1^ exhibited greater intensity, which may indicate a change in the binding state of water, while the overall reduction in band intensity in the fingerprint region (1100–1000 cm^−1^) points to an initial disruption in the molecular order of the pectin chains.

In the films with total glycerol replacement (F3), the changes were more drastic. The O-H band (~3334 cm^−1^) became the broadest and exhibited the lowest intensity among the three samples. The absence of the hydrophilic plasticizer disrupts the hydrogen-bond network previously mediated by water, preventing the relaxation of the pectin molecule. The C–H peaks (~2923 cm^−1^ and ~2853 cm^−1^) reached their maximum intensity, driven by the prevalence of saturated hydrocarbon chains, such as palmitic acid, inherent in the triglyceride fraction of buriti oil [46]. A significant change occurred in the 1800–1600 cm^−1^ region, where the peak at ~1745 cm^−1^ exhibited the highest absolute intensity. This marked increase is attributed to the C=O stretching vibrations in triglyceride esters and phenolic acids in buriti oil, which, now acting as the sole plasticizing component, overlap substantially with the remaining ester groups of the polymeric matrix [46]. This sharp increase in the intensity of C-H and C=O bands is expected to BO-added film, as occurred in other polymeric matrices described by Freitas et al. [22].

The band at ~1639–1622 cm^−1^ became relatively more prominent, suggesting a complex rearrangement of interactions and possibly of the state of water. The lower overall intensity in the fingerprint region indicates that exclusive plasticization by the lipid could promote a miscible molecular environment that is unfavorable for pectin chain vibrations. At the molecular level, the reduction in these stabilizing hydrogen bonds could indicate a thermodynamic incompatibility between the hydrophobic domains of the oil and the polar chains of pectin, which could be associated with the structural collapse, excessive porosity, and severe cracking observed in scanning electron microscopy (SEM) [36].

An analysis of the SSP polymer-based films revealed specific spectral and conformational dynamics in response to the progressive change in plasticizer. In the control film F1, the broad O-H/N-H band at ~3290 cm^−1^ exhibited high intensity. This band reflects the Amide A vibration, resulting from the strong hydrogen bonds formed between the protein’s N-H groups and the O-H groups derived from glycerol and moisture [23]. The aliphatic C–H peaks at ~2925 and ~2854 cm^−1^ appeared more faintly compared to formulations F2 and F3, while the prominent Amide I band at ~1645 cm^−1^ and Amide II band at ~1540 cm^−1^ characterized the main structure of helianthin [23].

In the F2 spectrum, the slightly reduced intensity of the O-H/N-H band (~3290 cm^−1^), the increase in the C-H vibrational peaks at 2925 cm^−1^, related to interactions between protein and lipid molecules [30], and the emergence of a new vibration at ~3006 cm^−1^ indicated the effective incorporation of buriti oil emulsion. The band at ~3006 cm^−1^ is herein assigned to the =C–H (sp^2^ bond) stretching vibration of *cis*-olefinic double bonds found in unsaturated fatty acids, such as oleic, linoleic, and linolenic acids. Furthermore, the appearance of an intensely sharp peak at ~1745 cm^−1^ is strictly attributed to the C=O stretching of triglyceride ester bonds derived from the lipid. The attenuation of the O-H/N-H band and the broadening of the C-H peaks also occur in films with total glycerol substitution (F3), reflecting the exclusion of water-mediated hydrophilic interactions and the complete dominance of the saturated hydrocarbon fraction [46]. Interestingly, the peak at ~1745 cm^−1^ associated with the oil ester remained well-defined, though with lower spectral intensity than that of F2. This decrease, contrary to the increase in oil content, may indicate the establishment of direct and specific interactions between the oil and the hydrophobic domains of the protein-only matrix, altering the vibrational absorptivity of the C=O bond. Although the overall reduction in peaks in the fingerprint region suggests significant variation in the signals due to different intermolecular interactions, the global Amide I and II bands remained strong and apparently unchanged. This pattern demonstrates that, given the high non-polarity of the medium, protein is forced to rearrange its domains to encompass the emulsion, which provides a molecular explanation for the formation of the dense, cohesive, and stable microstructure previously described in the morphological evaluation by scanning electron microscopy (SEM).

Regarding the pectin/SSP composite films, the control formulation F1 exhibited the most intense broad O-H/N-H band among the samples in its class (~3290 cm^−1^) [23]. The symmetrical profile of this band indicates that water and the plasticizer mediate hydrogen bonds between the protein amides and the polysaccharide hydroxyl groups, acting as a stabilizing dielectric bridge. At the same time, the thermodynamic affinity of the blend is supported by long-range electrostatic forces between the guanidinium cations (SSP) and the carboxylate anions (pectin), which inhibit polymeric repulsion and ensure matrix compatibility. A crucial spectral observation in F1 was the absence of a sharp, pronounced peak corresponding to the pectin alkyl ester at ~1745 cm^−1^. This attenuation suggests that, in the presence of SSP and glycerol, the carboxylate and ester groups are heavily involved in electrostatic and hydrogen-bonding interactions, significantly altering their vibrational response. The presence of the protein was confirmed by the strong Amide I band (~1643 cm^−1^), which suggests the peptidic C=O stretching superimposed on the vibration of water adsorbed onto the polymers [25], in addition to the presence of N-H bending and C-N stretching in Amide II (~1544 cm^−1^) [23].

With the partial and total replacement by buriti oil emulsion (F2 and F3), the effective introduction of hydrophobic domains into the matrix was observed, evidenced by a substantial increase in the aliphatic C–H stretching bands (~2925 and ~2854 cm^−1^) and the appearance of the oil ester band at ~1745 cm^−1^. In formulation F3, the maximum intensity of these lipid bands coincided with the lowest intensity and widest width of the O-H/N-H band, confirming the absence of glycerol and reinforcing the material’s more nonpolar characteristics. However, the Amide I and II bands remained unaffected by the exclusive lipid plasticization, showing minimal variations in intensity and width, as clearly evident in the spectrum. During solvent evaporation (drying), the extended polymer chain of pectin acts as a rigid structural framework that physically confines the SSP and oil structures. It is this intrinsic intermolecular kinetic constraint that dampens the lipid immiscibility shock and prevents drastic failures, explaining why the blended film (F3) maintained morpblenhological integrity far superior to that of the isolated pectin F3 formulation under SEM.

In order to characterize the variations in the protein’s secondary structure—which often overlap in the primary spectra—the Amide I region (1710–1590 cm^−1^) of the SSP-based formulations was analyzed using the deconvolution technique (presented in Figures S1–S6, Supplementary Material) [23,34]. The quantitative determination of the conformational fractions was calculated using the area of the previously fitted peaks, an analytical criterion adopted to mitigate errors in conventional height-based measurements [47]. It is known that a higher proportion of β-sheet structures results in more compact and thermodynamically stable networks, whereas a predominance of α-helices or random coils characterizes relaxed arrangements that are more prone to denaturation [34].

The results presented in Table 3 indicate that, in films composed purely of SSP, the thermodynamic competition generated by the simultaneous presence of glycerol and oil in sample F2 may have disrupted the protein’s compactness, causing an abrupt decline in β-sheets (from 36.71% to 19.83%) in contrast to the expansion of the relaxed regions to 73.27%. This restructuring could be due to changes in interfacial pH in the polymer solution, associated with the kinetic effects of the competition between plasticizers [23,48]. However, upon complete plasticization with oil emulsion (SSP F3), the elimination of hydrophilicity prompted the protein to rearrange its nonpolar domains into an emulsion-encapsulating conformation, which restored and increased the proportion of β-sheets to 41.60%, confirming the high density observed by SEM.

Finally, a deconvolution analysis confirmed the significant role of pectin as a mechanical and protective support element. In the F2 blended samples, the polysaccharide support prevented the relaxation that had occurred in the purely protein matrix, maintaining the proportion of β-sheets at 37.52%. The arrangement with the greatest structural cohesion was observed in the composite plasticized strictly by the lipid component (F3), where β-sheets achieved the highest computed score (44.65%), coupled with the maximum reduction in α-helix/random coil fractions (43.56%). This measurement of peak area demonstrates that the restrictive framework of pectin harmonizes with the plasticity of SSP to encapsulate the highly lipid-rich domains, resulting in matrices with a high degree of intramolecular packing and high structural cohesion.

### 3.4. Mechanical Properties

In pectin-based films, the mechanical properties showed significant variation depending on the plasticizer used, as shown in Table 4. For the control film, the results already indicated a mechanically balanced material with good strength and deformability. Results are similar to those reported by Qin et al. [49], that is, tensile strength (TS) values close to 20 MPa and elongation close to 5%.

When 50% of the glycerol was replaced with buriti oil emulsion (F2), a slight reduction in TS was observed, without significant difference, as well as a marked decrease in EAB, indicating that the incorporation of lipids and the replacement of the plasticizer compromised the film’s flexibility. The modulus of elasticity also increased significantly, suggesting even greater structural rigidity, possibly resulting from the reorganization of the polymer chains upon replacement of glycerol. In the formulation with 100% replacement of the plasticizer (F3), mechanical performance was substantially impaired. TS and EAB decreased dramatically, and it was not possible to calculate the modulus of elasticity. This behavior has already been reported in the literature by Spinei, Oroian, and Ursachi [31], who noted that, in some cases, vegetable oils can impart greater stiffness to the film by interfering with polymer mobility, likely due to low compatibility between the oil and the hydrophilic structure of pectin, with possible immobilization of the oil within the polymer matrix. It is important to highlight the severity of this loss of pectin performance when compared to other polysaccharide matrices. In previous studies, galactomannan films in which the hydrophilic plasticizer was completely replaced with the same emulsified buriti oil showed only an increase in stiffness, without structural failure [22].

The decline in pectin’s mechanical performance, reflected in the drastic drops in TS and EAB, is a direct macroscopic consequence of the cracks revealed by SEM. As evidenced by FTIR, the limiting thermodynamic incompatibility between the polar polysaccharide and the oil adversely affected the intermolecular interactions vital for stress transfer, thereby weakening the material.

The group of films composed solely of sunflower seed protein (SSP) exhibited the most distinct profile. The control, plasticized only with glycerol (F1), displayed higher tensile strength and elongation at break, along with a low modulus of elasticity, indicating a high capacity for elastic deformation. This highly plastic behavior may be related to the structural flexibility of the protein matrix in the presence of glycerol, which acts as a potent reducer of intermolecular stresses [50].

The partial replacement of the plasticizer (F2) reduced both strength and elongation and increased the elastic modulus (EM), leading to stiffer and less elastic behavior. In sample F3, however, there was a marked loss of performance, with drastic decreases in tensile strength (TS) and elongation at break (EAB), and again, the modulus could not be calculated. The same was also reported by Orliac et al. [32] when adding propylene glycol as a plasticizer to their sunflower seed protein-based films; they observed that the presence of a hydrophobic group in the plasticizer resulted in a specific orientation of the molecules within the protein structure, causing the film to become more brittle as the amount of plasticizer increased.

Finally, in the composite films, comprising pectin/SSP, the control (F1) proved to be a moderately strong and flexible material. The presence of the protein matrix promotes the formation of structured networks via hydrogen bonds and internal hydrophobic interactions, which provides good mechanical strength [50]. In the formulation with 50% glycerol (F2), TS remained stable while EAB was severely reduced and the modulus of elasticity increased, indicating that the oil emulsion acted as a stiffening agent, drastically reducing the film’s deformability. This phenomenon may be related to the reduction in bonds between protein chains and the formation of lipid microdomains that impair the continuity of the matrix [51].

In contrast, with total substitution by oil emulsion (F3), the film exhibited extremely reduced mechanical performance, once again making it impossible to calculate the modulus of elasticity, reflecting an almost total loss of mechanical integrity. This behavior can be attributed to the immiscibility between the oil and the matrix, which is predominantly protein-based. The presence of pectin, acting as a confining agent for the protein matrix, may also be one of the reasons for this behavior, as well as the specific properties of buriti oil and sunflower seed protein. As reported by other authors [50], different oils interact in different ways with each protein, directly modifying the mechanical properties of the films and leading to the formation of brittle and discontinuous films.

This impaired mechanical response resembles the behavior previously reported in complex matrices, such as pure starch and starch-galactomannan blends, in which the complete removal of glycerol also caused an abrupt drop in strength and elastic modulus [22]. As observed in starch-based mixtures, the low dispersion of lipid droplets in the matrix and the absence of a hydrophilic plasticizer resulted in a discontinuous and brittle network. This pattern reinforces the hypothesis that buriti oil emulsion, although it has functional potential, does not behave as an effective plasticizer when used alone in the tested matrices. On the other hand, the use of BO emulsion as a partial substitute for glycerol did not significantly change the TS of the films containing pectin, but increased the EM of all formulations. Therefore, the incorporation of BO can be a promising approach to reducing the use of petrochemical-based plasticizers. Anjos et al. [21] explained that the incorporation of BO in protein-based films containing glycerol can lead to a decrease in TS due to its polyphenol content that may facilitate protein–protein interactions. Similarly, Gomes et al. [16] showed that the addition of eugenol oil to SSP films also resulted in a decrease in TS and EM, with no significant changes in EAB, indicating that the results obtained in the present study are consistent with those generally reported for the addition of vegetable oils into biopolymeric films.

Furthermore, in a previous study [22], we observed that the use of gum arabic as an emulsifier may have impaired the action of buriti oil as a plasticizer. This behavior can be attributed to hydrogen-bond interactions between the polysaccharide matrix and the emulsifier layer surrounding the oil droplets, resulting in an anti-plasticizing effect. In light of this, we suggest conducting further tests using other homogenization techniques or alternative emulsifiers, as recommended by Tirgarian, Farmani, and Milani [51] when discussing the addition of different vegetable oils to protein films.

Although SEM revealed a visually cohesive and stable protein matrix for the films containing buriti oil, they became stiffer and less elastic in the absence of glycerol. This is explained by the secondary structures of the protein films observed in the FTIR spectra, manifested by excessive intramolecular packing (increased β-sheets), generating a highly interconnected and dense network that, lacking the lubrication provided by a hydrophilic plasticizer, loses chain mobility, culminating in brittle failure under tensile stress.

### 3.5. Thermogravimetric Analysis (TGA)

**Figure 8:**
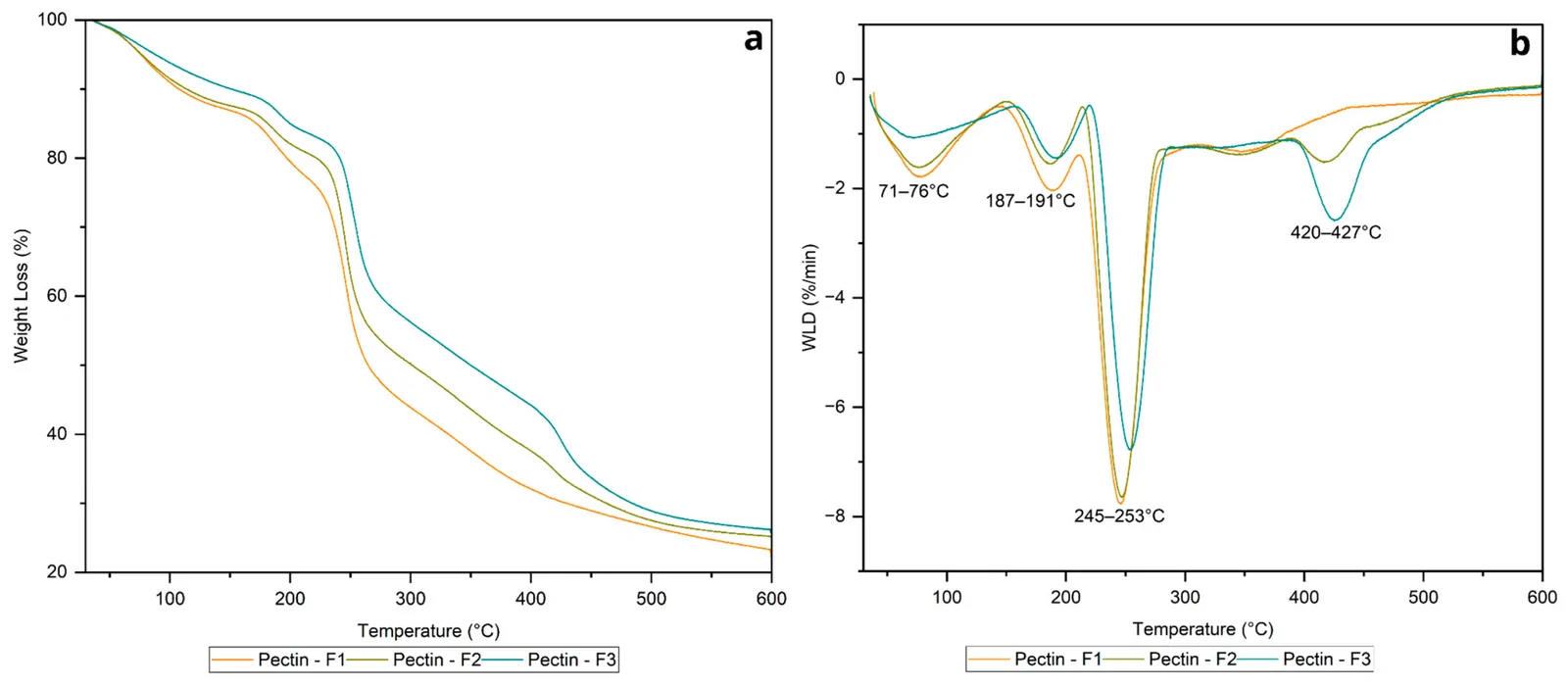
(**a**) Weight loss curves and (**b**) their derivatives for Pectin films.

**Figure 9:**
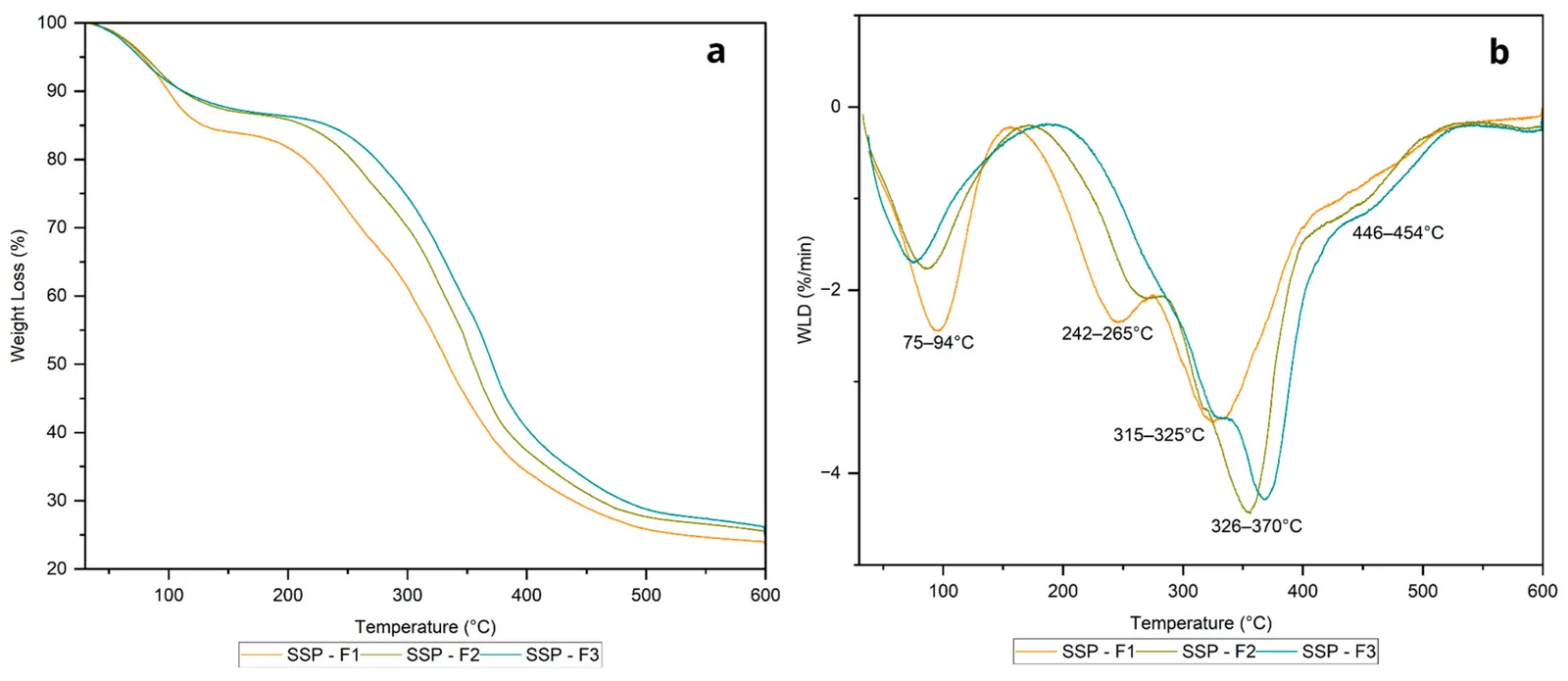
(**a**) Weight loss curves and (**b**) their derivatives for SSP films.

**Figure 10:**
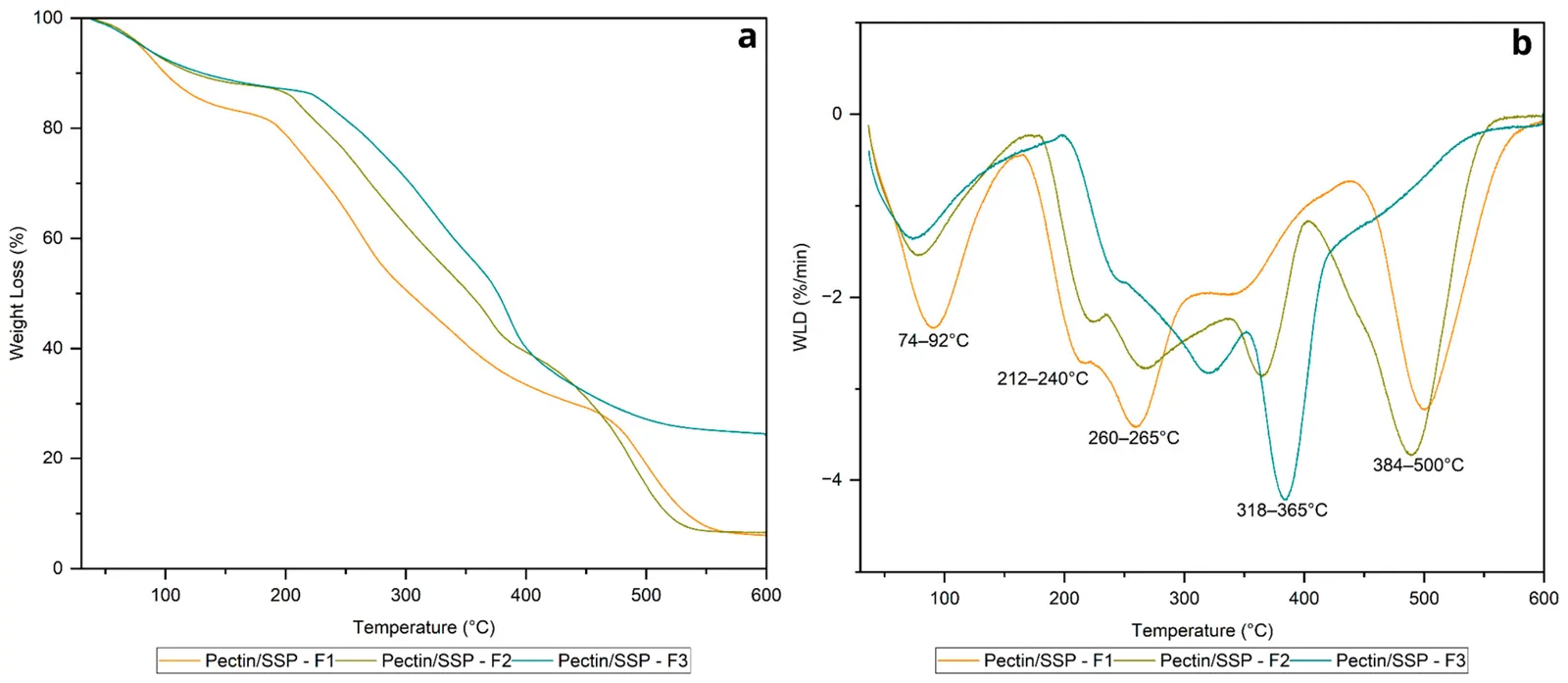
(**a**) Weight loss curves and (**b**) their derivatives for Pectin/SSP films.

The thermal stability and decomposition profile of films based on pectin, sunflower seed protein (SSP), and their blends, formulated with different proportions of glycerol and buriti oil, were analyzed by TGA/DTG, as shown in Figure 8, Figure 9 and Figure 10,, which revealed a thermal behavior characterized by multiple stages of mass loss.

The first stage of mass loss, with DTG peaks generally occurring below 100 °C, corresponds to the evaporation of water that is adsorbed and weakly bound to the film matrix [52]. In general, it was observed that the presence of glycerol resulted in greater mass losses in the moisture-related stage, which is consistent with the hygroscopic nature of this plasticizer. The full incorporation of buriti oil emulsion (F3) led to a lower mass loss in the first stage in all matrices, suggesting that the replacement of the hydrophilic plasticizer with a lipid component confers a nonpolar character to the films’ microstructure, in which the reduction of glycerol-mediated hydrogen bonds limited water retention within the material, reducing the polymer network’s ability to adsorb water from the environment [31]. A second thermal event, evidenced by peaks or shoulders on the DTG curves (generally between 180 °C and 265 °C), was attributed to the decomposition of glycerol in the samples containing it. Literature data indicate that this stage of glycerol decomposition can occur in the range of 170–250 °C [43,52]. In formulations with total or partial replacement of glycerol, changes in this stage were observed, with DTG peaks indicating the decomposition or volatilization of lighter components of buriti oil.

The main stage of biopolymer degradation occurred at higher temperatures, with intense DTG peaks. For pectin films, the maximum degradation temperature (T_max_) ranged from 245 °C (F1) to 252 °C (F3). Following this main event, another decomposition peak was observed in the DTG for the pectin films, occurring between 420 °C (F2) and 427 °C (F3). This stage at higher temperatures, especially in samples F2 and F3 containing buriti oil, can be attributed to the decomposition of more stable fractions of the oil and/or to a slower, stepwise carbonization of the pectin structure modified by the presence of the oil. Data from the literature indicate that cellulose/pectin films may undergo a primary degradation stage between 260 and 350 °C, and the addition of oils can influence thermal stability [31].

Films containing protein, whether pure or blended with pectin, exhibited an additional peak or a pronounced shoulder on the DTG curve, preceding the temperature of maximum degradation of the main protein structure. This intermediate thermal event, not observed with the same clarity in the other films, suggests a stepwise decomposition process for the SSP. Previous studies have shown that the decomposition of protein films can occur with multiple peaks on the DTG curve [53]. This reflects the heterogeneous nature of the protein isolate, which contains different fractions such as globulins and albumins, and the sequential degradation of secondary and tertiary structures prior to the complete fragmentation of the main polypeptide chain.

In pure SSP films, the main protein degradation occurred with T_max_ ranging from 326 °C (F1) to 370 °C (F3). The PISG samples with partial (F2) and total (F3) oil emulsion substitution also exhibited a final, faint peak in the DTG curve corresponding to decomposition at very high temperatures, between 446 and 454 °C. These events observed in the SSP films containing oil may be related to the decomposition of different fractions of buriti oil or to specific oil-protein interactions that result in products with different thermal stabilities, in addition to protein carbonization.

The thermal profile of the pectin/SSP composites was distinctly influenced by the composition of the plasticizer, with the degradation of the polymeric components (SSP and pectin) occurring in multiple stages, particularly evident before the final stage of carbonization. In the samples containing glycerol (F1 and F2), most of the polymer decomposition occurred in two or three main stages, between approximately 260 °C and 365 °C, followed by a well-defined final carbonization peak around 490 and 500 °C and significant mass losses, possibly due to the way glycerol interacts with the matrix and influences the formation of carbonaceous residues that decompose later [52]. In contrast, in the Pectin/SSP composite with 100% oil emulsion (F3), the absence of glycerol and the high concentration of buriti oil resulted in a different profile: a degradation event, suggesting a joint decomposition of the polymers and the oil, and a carbonization event, with lower mass loss in this final stage and a lower temperature at which the highest degradation rate occurs, compared to the final stages of F1 and F2. This suggests that the type of plasticizer (glycerol vs. buriti oil emulsion) alters not only the initial stages but also the mechanism and final degradation temperatures of the Pectin/SSP composites.

In summary, the addition of buriti oil emulsion influenced the degradation profile of pectin films, SSP films, and their blends, introducing or modifying decomposition stages, especially at higher temperatures. With regard to thermal stability, although variations were observed in the main polymers depending on the matrix and formulation, the mass loss curves reveal a consistent increase in film stability when glycerol was replaced by buriti oil emulsion, with the exception of the blended films. It is interesting to note the contrast between this behavior and that of other polymeric matrices. While the addition of the emulsified lipid did not significantly alter the maximum primary degradation temperature in starch and galactomannan films [22], the present study reveals that the oil acts as an effective thermal retarder for pectin and isolated protein.

### 3.6. Moisture Content (MC) and Water Solubility (WS)

With regard to the moisture content of the films, as shown in Table 5, a general trend towards decrease was observed with the addition of buriti oil emulsion. Because buriti oil is nonpolar, it hinders water retention in the matrix, in contrast to glycerol, which is highly hygroscopic [6].

As to water solubility (see Table 5), distinct behaviors were observed for each polymer matrix. The pectin films exhibited high solubility, with no statistically significant differences between the control and the films with added buriti oil emulsion. This result corroborates the observation regarding thickness, suggesting that pectin, with its greater affinity for polar compounds, limits the effective integration of hydrophobic constituents such as buriti oil into the matrix, thereby maintaining its high hydrophilicity [23,54]. This behavior contrasts with that of other polysaccharides previously evaluated [22], in which the addition of the same emulsified buriti oil was sufficient to drastically reduce the water solubility of polysaccharide matrices, leading to a significant increase in the hydrophobicity of the network.

On the other hand, the protein films showed a clear reduction in solubility with the incorporation of the oil, indicating the formation of a more hydrophobic network due to the interaction between the protein and the oil. However, the complete replacement of glycerol in formulation F3 of the pure protein films resulted in an increase in solubility compared to formulation F2, although this value remained significantly lower than that observed in the control sample. The trend toward increased solubility in F3, following the marked reduction observed in F2, can be attributed to the saturation of the protein matrix by the lipid component, promoting a conformational reorganization that favors the exposure of hydrophilic functional groups. This behavior is corroborated by the analysis of the secondary structures of the Amide I band, which revealed a significant increase in β-sheet structures in the F3 film, suggesting that the matrix adopted a rigid conformation, making it difficult for its polar domains to be hidden during folding. Alternatively, this behavior may reflect limited compatibility among the formulation’s constituents at high oil concentrations [55].

In the pectin/SSP blends, oil incorporation led to a significant reduction in solubility compared to the control film, although F2 and F3 did not differ statistically from one another. This suggests that the presence of the protein in the blended films favored oil incorporation, resulting in films with lower water solubility that act as an effective barrier to moisture, as observed by other authors for protein films [29,32].

A comparative analysis of the different polymer groups reinforced that films containing sunflower seed protein (SSP) were more sensitive to plasticizer substitution, exhibiting the greatest variations in both solubility and moisture content. This may be related to the high reactivity of the peptide chains, which promote structural reorganization upon the introduction of hydrophobic plasticizers [56]. In contrast, pectin films exhibit more stable behavior, possibly due to the rigid linear structure resulting from interactions between pectin chains that promote strong intermolecular bonds, which may hinder oil penetration or interference within the matrix [5].

This greater responsiveness of films containing sunflower seed protein can be attributed to the amphiphilic nature and more complex structure of proteins compared to polysaccharides such as pectin. Proteins possess a variety of amino acid side chains with different polarities, which can establish diverse interactions (hydrophobic, hydrophilic, ionic) with buriti oil. During the solubilization of the polymer at pH 11, while the film is forming, these groups are exposed as the molecule unfolds, and the subsequent reorganization in the presence of a lipid component can lead to a structural arrangement significantly different from that obtained with a polar plasticizer such as glycerol, influencing both the thickness and the water-retention capacity of the film formed thereof [55].

The reduction in water solubility and moisture content with the full incorporation of buriti oil emulsion highlights the hydrophobic character imposed on the network. Especially in films containing SSP, the strong structural compaction (rich in β-sheets) and the morphological cohesion observed by SEM act synergistically to limit solvent penetration. The matrix has less free volume and fewer exposed hydrophilic sites, hindering the hydration of the polymer chains.

### 3.7. Barrier Properties

Replacing glycerol with buriti oil resulted in significant changes in the barrier properties of the films produced with pectin, sunflower seed protein, and their composites. In general, it was observed that the incorporation of the oil had a more pronounced effect on water vapor permeability (WVP) and water vapor transmission rate (WVTR), while the effects on oxygen permeability (OXP) varied depending on the matrix composition, as shown in Table 6.

In films prepared with pectin, the addition of oil resulted in a significant reduction in WVTR (for both films) and WVP (for the pure pectin film), indicating that the presence of buriti oil contributed to a more hydrophobic structure that was less permeable to water vapor, likely due to the formation of lipid domains that hinder the diffusion of water molecules through the film matrix—an effect that was even more pronounced in the mixed matrix, likely through hydrophobic interactions between the protein and lipid components [54].

However, the OP values in films produced with pectin alone did not show statistically significant differences among the samples, suggesting that the modification of the matrix was not sufficient to significantly restrict the transport of nonpolar gases such as oxygen. In contrast, in the composite films, OP was considerably reduced in formulation F3, with total glycerol replacement, suggesting that the combination of protein and oil favored the formation of a dense and cohesive network capable of hindering both water vapor and oxygen transport.

In films made exclusively from protein (SSP), the replacement of the plasticizer (total or partial) resulted in a more modest reduction in WVTR compared to the other matrices, even though the results still indicate a consistent trend toward improved moisture barrier performance with the incorporation of oil. Water vapor permeability (WVP) and oxygen permeability (OP) showed occasional variations but remained statistically unchanged across the different proportions of oil incorporated into the films [29].

It is important to note that proteins, on their own, are already recognized for forming films with good barrier properties, especially at low relative humidity, due to their compact structure and ability to form a dense network of hydrogen bonds [1,39]. The addition of buriti oil emulsion, in this case, appears not only to have contributed to improving the water vapor barrier, especially in films containing pectin, but also to have enhanced or complemented the oxygen barrier of the blended matrix.

### 3.8. Biodegradability

**Figure 11:**
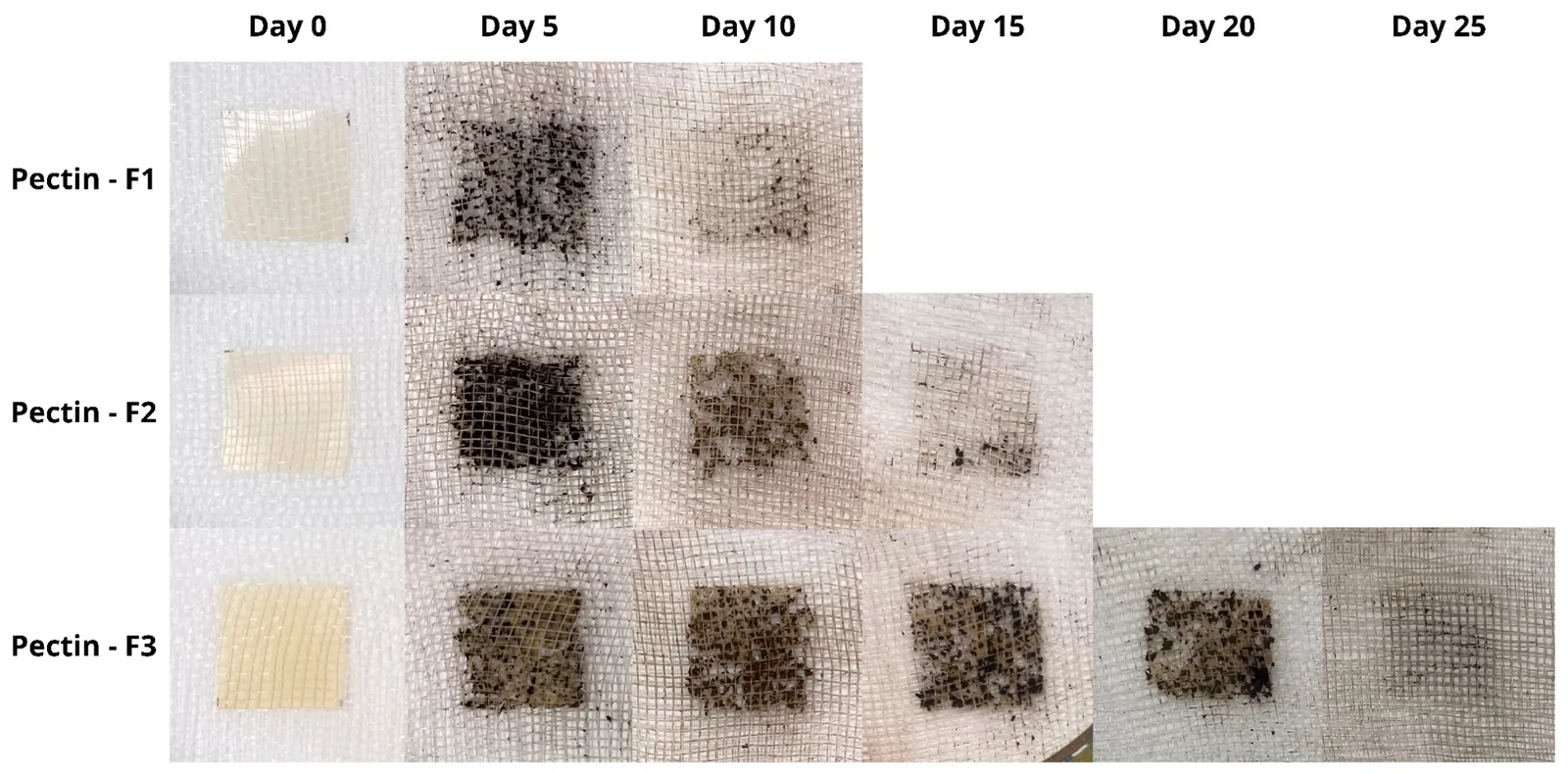
Biodegradability results for the pectin-based films.

**Figure 12:**
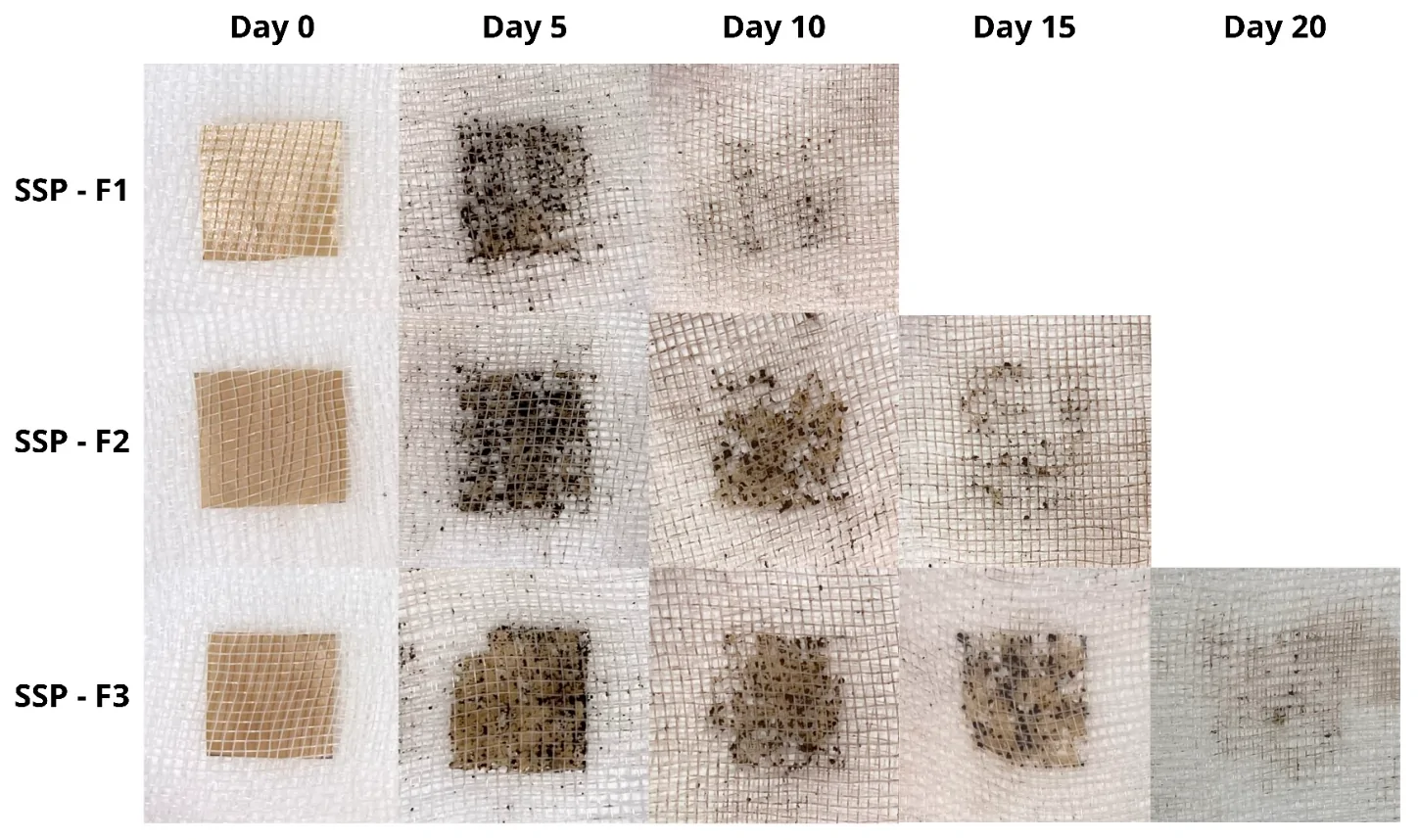
Biodegradability results for the SSP-based films.

**Figure 13:**
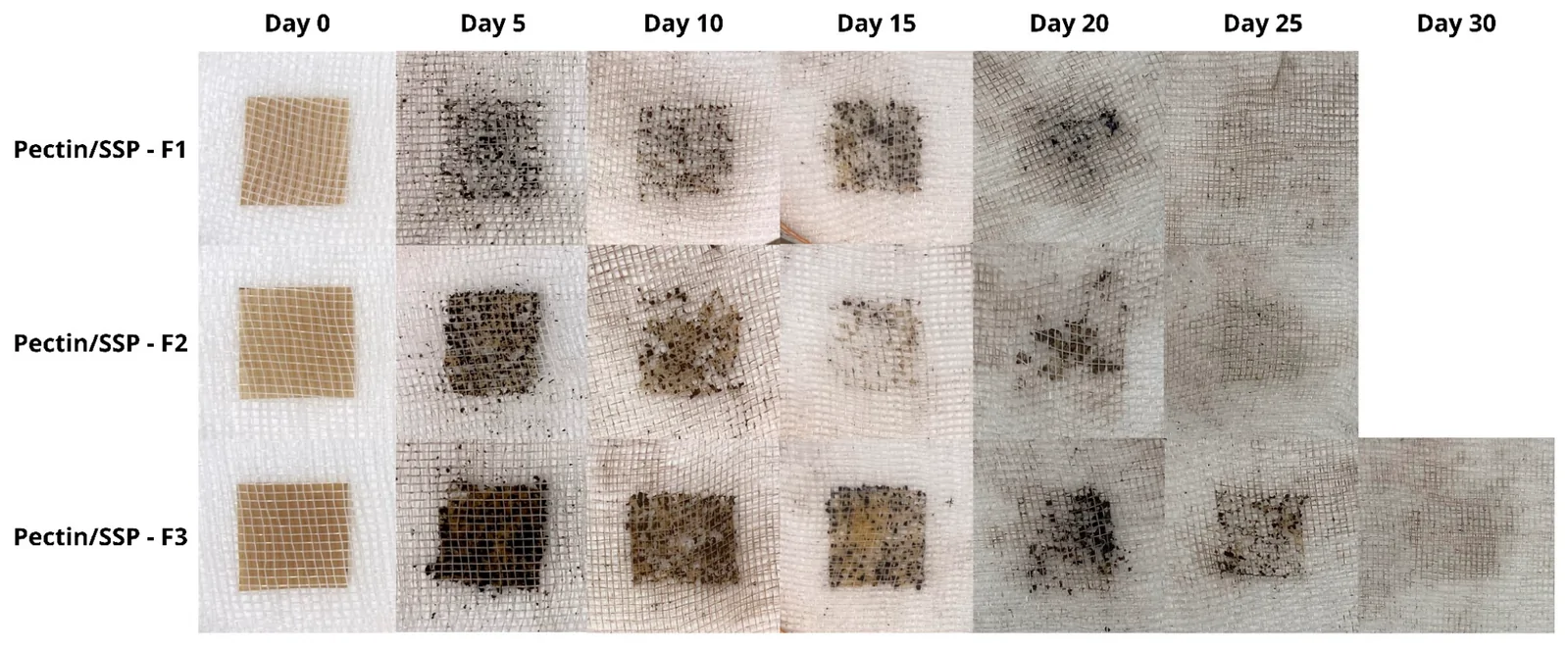
Biodegradability results for the blended films.

The biodegradability results for the films show that replacing glycerol with buriti oil emulsion directly influenced the maximum disintegration time, as can be seen in Figure 11, Figure 12 and Figure 13. The F1 control films, produced using only glycerol, exhibited low disintegration times, highlighting the greater ease of disintegration conferred by the hydrophilic nature of glycerol.

With the partial (F2) and total (F3) replacement of glycerol with buriti oil emulsion, a gradual increase in resistance to disintegration was observed in all films. This behavior is related to the oil’s hydrophobicity, which limits moisture absorption and microbial activity [57]. This direct correlation between lipid content and extended shelf life in soil confirms the disintegration pattern previously established for other matrices [22]. A comparison of the four biopolymers reveals a unique behavior: the increase in hydrophobicity caused by buriti oil acts to slow hydration and microbial attack.

Pure protein-based films showed a less pronounced response to increased oil content, with a maximum of 20 days, which may be related to the lower structural cohesion of the isolated protein matrix. In contrast, the pectin/SSP composites exhibited the greatest resistance, reaching up to 30 days of disintegration for films with total glycerol replacement (F3). This result suggests that combining protein with pectin promotes the formation of a more stable structure, confirming that polymer morphology directly affects biodegradation rates [58].

Buriti oil emulsion contributes to greater stability and durability of the material under environmental conditions that would otherwise promote rapid microbial action. This results in a more controlled biodegradation rate, which is noteworthy due to the necessity of biodegradable materials to end plastic pollution.

### 3.9. Comparative Analysis with Literature Data

A comparison between some of the results obtained in this study with other polymeric matrices, regarding physical, mechanical and barrier properties, is shown in Table 7. For this analysis, only the F2 formulations (partial replacement of the plasticizer with buriti oil emulsion) were selected, as these represented the best balance between structural integrity and barrier efficiency in our work. The suitability of buriti oil emulsion as a plasticizer and stabilizer was evaluated in different biopolymer matrices, including pectin and sunflower protein (SSP) films, both pure and in blends. In the pure pectin matrix, the oil exhibited high tensile strength (20.85 MPa), surpassing pectin matrices supplemented with lemon oil studied by Akachat et al. [11] (14.68 MPa) and approaching the tensile strength of the gelatine films with buriti oil developed by Anjos et al. [21] (18.68 MPa). In the pure SSP matrix, plasticization with the oil resulted in the highest elongation (29.48%) and drastically reduced water solubility to 17.89%, representing superior structural performance when compared to the brittleness and hydrophilicity of SSP films with clove oil described by Salgado et al. [29] (2.500 MPa and 86.3% solubility). The use of buriti oil also acted as a counterbalance to the stiffness of bioplastics such as pure PLA described by Mohammadi et al. [59], which exhibited an elongation of 4.95%. The blend (Pectin/SSP), in turn, functioned as an intermediate system, balancing strength (8.956 MPa) and solubility (36.47%).

With regard to barrier properties, the incorporation of buriti oil demonstrated strong hydrophobic capacity, reducing water vapor permeability (WVP) in all formulations, particularly in the blend (0.670 × 10^−10^ g/s·m·Pa) and pure pectin films (0.805 × 10^−10^ g/s·m·Pa). The most significant technological impact of this development, however, lies in the remarkable oxygen barrier properties of the tested matrices compared to synthetic polymers such as LDPE. The pure matrices were shown to possess a good intrinsic gas barrier (reaching 0.980 g·mm/m^2^·day·kPa for SSP and 1.123 g·mm/m^2^·day·kPa for pectin), which was not adversely affected by the addition of lipids. In stark contrast, fossil-based synthetic polymers, such as the LDPE studied by Patil et al. [60], effectively blocked moisture (0.169 g·mm/m^2^·day·kPa), but performed very poorly in terms of gas permeability (212.150 cm^3^·mm/m^2^·day). Therefore, whilst biopolymer matrices provide a better oxygen barrier than conventional plastics, buriti oil acts synergistically with glycerol, optimising the moisture resistance of the final material.

## 4. Conclusions

This study investigated the impact of buriti oil emulsion on the replacement of glycerol in citrus pectin and sunflower seed protein (SSP) films, both on its own and in composites. This evaluation broadens the scope of our study, allowing for a comparison with previously evaluated biopolymer systems. FTIR structural analysis demonstrated the different interactions resulting from the replacement of glycerol in varying proportions for each of the biopolymer matrices.

The mechanical properties were substantially compromised by the complete substitution of glycerol, suggesting that buriti oil emulsion, when used alone, did not function as an effective plasticizing agent for these matrices. In pectin-based films, though, pure or in blend, it was observed that the partial substitution did not significantly change TS property. However, a thermogravimetric analysis (TGA) indicated that the thermal stability of the pure pectin and SSP films increased when glycerol was replaced by buriti oil.

In terms of physicochemical characteristics, the inclusion of buriti oil generally resulted in lower moisture absorption and lower water solubility, particularly pronounced in systems rich in SSP. Regarding barrier properties, the added buriti oil emulsion resulted in similar performances for the majority of the analyzed films in terms of WVP and OP. Biodegradability was influenced by the presence of the oil emulsion, which prolonged the disintegration time compared to the control films, without losing their biodegradable nature.

In summary, buriti oil emulsion has proven to be a promising functional additive for optimizing MC, WS and thermal stability of pectin and SSP films. However, its use as the sole plasticizer requires future formulation optimizations to balance its benefits without compromising the structural integrity and flexibility of the final film.

## Figures and Tables

**Table 1:** Mass composition of the prepared films.

Polymer	Film	Pectin (g)	SSP (g)	Glycerol (g)	Buriti Oil (g)	Gum Arabic (g)	Water (mL)
**Pectin**	**F1**	3	-	0.6	-	-	196.4
	**F2**	3	-	0.3	0.3	0.09	196.3
	**F3**	3	-	-	0.6	0.18	196.2
**SSP**	**F1**	-	3	0.6	-	-	196.4
	**F2**	-	3	0.3	0.3	0.09	196.3
	**F3**	-	3	-	0.6	0.18	196.2
**Pectin/SSP (1:3)**	**F1**	0.75	2.25	0.6	-	-	196.4
	**F2**	0.75	2.25	0.3	0.3	0.09	196.3
	**F3**	0.75	2.25	-	0.6	0.18	196.2

(F1) control films; (F2) films with partial replacement of glycerol with buriti oil emulsion; (F3) films with total replacement of glycerol with buriti oil emulsion.

**Table 2:** Chromatic parameters and thickness of the films.

Polymer	Film	Color			Opacity (mm^−1^)	Thickness (mm)
		L^*^	c^*^	h^*^		
**Pectin**	**F1**	25.940 ± 1.511 ^c^	1.647 ± 0.372 ^b^	103.440 ± 5.790 ^a^	9.005 ± 0.197 ^b^	0.054 ± 0.004 ^a^
	**F2**	32.910 ± 0.513 ^b^	4.265 ± 1.087 ^b^	97.940 ± 4.240 ^ab^	8.387 ± 0.249 ^b^	0.069 ± 0.015 ^a^
	**F3**	37.410 ± 1.182 ^a^	10.420 ± 3.270 ^a^	91.250 ± 3.020 ^b^	17.958 ± 1.007 ^a^	0.053 ± 0.016 ^a^
**SSP**	**F1**	12.330 ± 1.980 ^c^	21.270 ± 1.990 ^a^	70.240 ± 4.540 ^b^	17.527 ± 0.902 ^a^	0.041 ± 0.005 ^b^
	**F2**	23.520 ± 0.455 ^a^	11.840 ± 0.226 ^b^	77.777 ± 1.381 ^a^	14.434 ± 0.665 ^b^	0.060 ± 0.010 ^a^
	**F3**	18.930 ± 2.070 ^b^	22.050 ± 2.67 ^a^	76.738 ± 1.438 ^ab^	13.827 ± 1.018 ^b^	0.065 ± 0.006 ^a^
**Pectin/SSP (1:3)**	**F1**	28.550 ± 1.770 ^a^	6.122 ± 0.828 ^b^	75.840 ± 5.040 ^a^	12.990 ± 0.847 ^a^	0.065 ± 0.003 ^c^
	**F2**	28.450 ± 2.700 ^a^	10.325 ± 0.709 ^ab^	76.017 ± 0.926 ^a^	12.223 ± 0.565 ^a^	0.074 ± 0.003 ^b^
	**F3**	24.190 ± 1.790 ^a^	13.020 ± 3.460 ^a^	74.080 ± 3.900 ^a^	12.309 ± 0.525 ^a^	0.089 ± 0.006 ^a^

(F1) control films; (F2) films with partial replacement of glycerol with buriti oil emulsion; (F3) films with total replacement of glycerol with buriti oil emulsion. Means with the same letters in the same column for a specific basis do not show statistical differences (*p* > 0.05).

**Table 3:** Deconvolution data for the secondary protein structure region of the blended and SSP-based films.

Polymer	Film	β-Sheet (%)	α-Helix/Random Coils (%)	β-Turn (%)	Antiparallel β-Sheet (%)
**SSP**	**F1**	36.7109	57.1810	4.1176	1.9914
	**F2**	19.8314	73.2755	4.9704	1.9228
	**F3**	41.6050	49.9807	5.3815	3.0329
**Pectin/SSP (1:3)**	**F1**	37.5475	59.3482	0.5007	2.6036
	**F2**	37.5225	51.7675	8.8025	1.9075
	**F3**	44.6504	43.5621	10.2623	1.5253

(F1) control films; (F2) films with partial replacement of glycerol with buriti oil emulsion; (F3) films with total replacement of glycerol with buriti oil emulsion.

**Table 4:** Mechanical properties of the films.

Polymer	Film	Tensile Strength (TS) (MPa)	Elongation at Break (EAB) (%)	Elasticity Modulus (EM) (MPa)
**Pectin**	**F1**	22.291 ± 2.226 ^a^	19.550 ± 2.169 ^a^	266.800 ± 41.600 ^b^
	**F2**	20.850 ± 6.830 ^a^	10.515 ± 2.188 ^b^	373.800 ± 95.500 ^a^
	**F3**	9.840 ± 5.720 ^b^	4.073 ± 1.710 ^c^	-
**SSP**	**F1**	6.456 ± 0.840 ^a^	157.000 ± 27.900 ^a^	28.870 ± 4.150 ^b^
	**F2**	4.418 ± 0.408 ^b^	29.480 ± 5.930 ^b^	73.290 ± 8.410 ^a^
	**F3**	1.700 ± 1.513 ^c^	1.171 ± 0.642 ^c^	-
**Pectin/SSP (1:3)**	**F1**	8.298 ± 0.606 ^a^	34.889 ± 1.848 ^a^	97.010 ± 12.570 ^b^
	**F2**	8.956 ± 1.641 ^a^	9.321 ± 2.120 ^b^	192.800 ± 36.700 ^a^
	**F3**	0.350 ± 0.225 ^b^	1.747 ± 1.097 ^c^	-

(F1) control films; (F2) films with partial replacement of glycerol with buriti oil emulsion; (F3) films with total replacement of glycerol with buriti oil emulsion. Means with the same letters in the same column for a specific basis do not show statistical differences (*p* > 0.05).

**Table 5:** Moisture content and solubility in water of the films.

Polymer	Film	MC (%)	WS (%)
**Pectin**	**F1**	15.790 ± 2.610 ^a^	86.830 ± 3.230 ^a^
	**F2**	13.187 ± 0.025 ^ab^	82.400 ± 5.020 ^a^
	**F3**	10.637 ± 0.277 ^b^	83.408 ± 0.543 ^a^
**SSP**	**F1**	13.960 ± 2.920 ^a^	42.960 ± 2.440 ^a^
	**F2**	8.294 ± 0.516 ^b^	17.890 ± 3.380 ^c^
	**F3**	8.030 ± 2.490 ^b^	29.050 ± 1.830 ^b^
**Pectin/SSP** **(1:3)**	**F1**	13.533 ± 0.925 ^a^	50.610 ± 5.340 ^a^
	**F2**	7.996 ± 0.629 ^b^	36.470 ± 3.740 ^b^
	**F3**	8.741 ± 0.200 ^b^	38.420 ± 4.150 ^b^

(F1) control films; (F2) films with partial replacement of glycerol with buriti oil emulsion; (F3) films with total replacement of glycerol with buriti oil emulsion. Means with the same letters in the same column for a specific basis do not show statistical differences (*p* > 0.05).

**Table 6:** Barrier properties of the films.

Polymer	Film	WVTR (×10^3^ g/s·m^2^)	WVP (×10^10^ g/s·m·Pa)	OP (g·mm/m^2^·day·kPa)
**Pectin**	**F1**	4.993 ± 0.315 ^a^	1.529 ± 0.098 ^a^	0.909 ± 0.176 ^a^
	**F2**	2.048 ± 0.292 ^b^	0.805 ± 0.115 ^b^	1.123 ± 0.023 ^a^
	**F3**	2.021 ± 0.638 ^b^	0.612 ± 0.193 ^b^	0.916 ± 0.162 ^a^
**SSP**	**F1**	4.798 ± 0.224 ^a^	1.173 ± 0.052 ^a^	1.267 ± 0.026 ^a^
	**F2**	3.443 ± 0.239 ^b^	1.161 ± 0.080 ^a^	0.980 ± 0.161 ^a^
	**F3**	3.211 ± 0.197 ^b^	1.348 ± 0.278 ^a^	1.150 ± 0.249 ^a^
**Pectin/SSP (1:3)**	**F1**	3.160 ± 0.624 ^a^	1.171 ± 0.231 ^a^	1.916 ± 0.073 ^a^
	**F2**	1.588 ± 0.528 ^b^	0.670 ± 0.222 ^a^	2.060 ± 0.065 ^a^
	**F3**	1.593 ± 0.510 ^b^	0.812 ± 0.260 ^a^	1.226 ± 0.216 ^b^

(F1) control films; (F2) films with partial replacement of glycerol with buriti oil emulsion; (F3) films with total replacement of glycerol with buriti oil emulsion. Means with the same letters in the same column for a specific polymeric matrix do not show statistical differences (*p* > 0.05).

**Table 7:** Mechanical and barrier properties of pectin and sunflower protein (SSP) matrices containing buriti oil, compared with reference materials.

Filmogenic Matrix	Tensile Strength (TS) (MPa)	Elongation at Break (EAB) (%)	Elasticity Modulus (EM) (MPa)	Water Solubility (WS) (%)	Water Vapor Permeability (WVP)	Oxygen Permeability (OP/OTR)	Reference
**Pectin + Glycerol + Buriti oil**	20.850 ± 6.830	10.515 ± 2.188	373.800 ± 95.500	82.400 ± 5.020	0.805 ± 0.115 (×10^−10^ g/s·m·Pa)	1.123 ± 0.023 (g·mm/m^2^·day·kPa)	
**SSP + Glycerol + Buriti oil**	4.418 ± 0.408	29.480 ± 5.930	73.290 ± 8.410	17.890 ± 3.380	1.161 ± 0.080 (×10^−10^ g/s·m·Pa)	0.980 ± 0.161 (g·mm/m^2^·day·kPa)	
**Pectin/SSP + Glycerol + Buriti oil**	8.956 ± 1.641	9.321 ± 2.120	192.800 ± 36.700	36.470 ± 3.740	0.670 ± 0.222 (×10^−10^ g/s·m·Pa)	2.060 ± 0.065 (g·mm/m^2^·day·kPa)	
**Pectin + Citric Acid + Glycerol + Lemon Oil**	14.680 ± 1.419	20.050 ± 0.784	174.730 ± 1.915	16.046	Not reported	Not reported	[11]
**Sunflower protein concentrate + glycerol + clove oil**	2.500 ± 0.200	24.900 ± 1.700	0.250 ± 0.030	86.300 ± 1.400	1.160 ± 0.090 (×10^−10^ g_H2O_/Pa·m·s)	Not reported	[29]
**Gelatine + Glycerol + Acetic acid + Buriti oil**	18.680 ± 6.780	8.380 ± 2.260	232.900 ± 87.640	Not reported	16.940 ± 2.130 (g_H2O_·mm/dm^2^·kPa)	Not reported	[21]
**Poly(lactic acid) (PLA)**	46.920 ± 1.570	4.950	Not reported	Insoluble	3.398 ± 0.110 (×10^−8^ gm^−2^·24 h^−1^·Pa^−1^)	Not reported	[59]
**PLA + Dioctyl terephthalate (DOTP) + clove essential oil**	39.220 ± 1.810	44.010	Not reported	Insoluble	4.149 ± 0.180 (×10^−8^ gm^−2^·24 h^−1^·Pa^−1^)	Not reported	[59]
**Low-Density Polyethylene (LDPE)**	15.100 ± 1.710	312.910 ± 15.750	0.460 ± 0.120	0.000 ± 0.000	0.169 ± 0.030 (g·mm/m^2^·day·kPa)	212.150 ± 1.230 (cm^3^·mm/m^2^·day)	[60]

## Data Availability

The original contributions presented in the study are included in the article/Supplementary Material; further inquiries can be directed at the corresponding author.

## Supplementary Materials

The following supporting information can be downloaded at https://www.mdpi.com/article/10.3390/foods15213779/s1. Table S1. Statistical parameters and goodness-of-fit for the mathematical deconvolution of the Amide I region (1600–1700 cm^−1^) in the FTIR spectra of the films. Figure S1: FTIR amide I peak deconvolution of SSP-F1; Table S2. Peak assignments and relative areas of secondary structures obtained from the mathematical deconvolution of the Amide I region (1600–1700 cm^−1^) for the SSP-F1 film. Figure S2: FTIR amide I peak deconvolution of SSP-F2; Table S3. Peak assignments and relative areas of secondary structures obtained from the mathematical deconvolution of the Amide I region (1600–1700 cm^−1^) for the SSP-F2 film. Figure S3: FTIR amide I peak deconvolution of SSP-F3; Table S4. Peak assignments and relative areas of secondary structures obtained from the mathematical deconvolution of the Amide I region (1600–1700 cm^−1^) for the SSP-F3 film. Figure S4: FTIR amide I peak deconvolution of Pectin/SSP-F1; Table S5. Peak assignments and relative areas of secondary structures obtained from the mathematical deconvolution of the Amide I region (1600–1700 cm^−1^) for the Pectin/SSP-F1 film. Figure S5: FTIR amide I peak deconvolution of Pectin/SSP-F2; Table S6. Peak assignments and relative areas of secondary structures obtained from the mathematical deconvolution of the Amide I region (1600–1700 cm^−1^) for the Pectin/SSP-F2 film. Figure S6: FTIR amide I peak deconvolution of Pectin/SSP-F3. Table S7. Peak assignments and relative areas of secondary structures obtained from the mathematical deconvolution of the Amide I region (1600–1700 cm^−1^) for the Pectin/SSP-F3 film.

## Abbreviations

The following abbreviations are used in this manuscript:

BO	Buriti oil
DCA	Departamento de Ciência de Alimentos
DEMEC	Departamento de Engenharia Mecânica
EM	Elasticity Modulus
FTIR	Fourier Transform Infrared
EAB	Elongation at Break
MC	Moisture content
OXP	Oxygen permeability
PPGCA	Programa de Pós-Graduação em Ciência de Alimentos
SEM	Scanning electron microscopy
SSP	Sunflower seed protein
TGA	Thermogravimetric analysis
TS	Tensile strength
WLD	Weight loss derivative
WS	Water solubility
WVP	Water vapor permeability
WVTR	Water vapor transmission rate

## References

[1] Suderman N., Isa M.I.N., Sarbon N.M. (2018). The Effect of Plasticizers on the Functional Properties of Biodegradable Gelatin-Based Film: A Review. Food Biosci..

[2] Cheng J., Gao R., Zhu Y., Lin Q. (2024). Applications of Biodegradable Materials in Food Packaging: A Review. Alex. Eng. J..

[3] Das Adhikary N., Bains A., Sridhar K., Kaushik R., Chawla P., Sharma M. (2023). Recent Advances in Plant-Based Polysaccharide Ternary Complexes for Biodegradable Packaging. Int. J. Biol. Macromol..

[4] Dejene B.K. (2025). Eco-Friendly Synthesis of Metallic Nanoparticles from Agri-Food Waste Extracts: Applications in Food Packaging and Healthcare–A Critical Review. Mater. Today Chem..

[5] Butler I.P., Banta R.A., Tyuftin A.A., Holmes J., Pathania S., Kerry J. (2023). Pectin as a Biopolymer Source for Packaging Films Using a Circular Economy Approach: Origins, Extraction, Structure and Films Properties. Food Packag. Shelf Life.

[6] Syarifuddin A., Muflih M.H., Izzah N., Fadillah U., Ainani A.F., Dirpan A. (2025). Pectin-Based Edible Films and Coatings: From Extraction to Application on Food Packaging towards Circular Economy- A Review. Carbohydr. Polym. Technol. Appl..

[7] Kaur R.P., Ghoshal G. (2022). Sunflower Protein Isolates-Composition, Extraction and Functional Properties. Adv. Colloid Interface Sci..

[8] Perera K.Y., Jaiswal A.K., Jaiswal S. (2023). Biopolymer-Based Sustainable Food Packaging Materials: Challenges, Solutions, and Applications. Foods.

[9] Sun S., Weng Y., Zhang C. (2024). Recent Advancements in Bio-Based Plasticizers for Polylactic Acid (PLA): A Review. Polym. Test..

[10] Farhan A., Fazial F.F., Azfaralariff A., Costa M.J., Cerqueira M.A. (2025). Production of Polysaccharide and Protein Edible Films: Challenges and Strategies to Scale-Up. Int. J. Biol. Macromol..

[11] Akachat B., Himed L., Salah M., D’Elia M., Rastrelli L., Barkat M. (2025). Development of Pectin-Based Films with Encapsulated Lemon Essential Oil for Active Food Packaging: Improved Antioxidant Activity and Biodegradation. Foods.

[12] Liu Z., Yang M., Hu L., Xu J., Wang F., Liu L., Kang J. (2025). Biodegradable Active Films Developed from Low-Methoxyl Pectin Incorporated with Cellulose Nanocrystals-Stabilized Oregano Essential Oil Pickering Emulsions. Int. J. Biol. Macromol..

[13] Nahas E.O., Furtado G.F., Lopes M.S., Silva E.K. (2025). From Emulsions to Films: The Role of Polysaccharide Matrices in Essential Oil Retention Within Active Packaging Films. Foods.

[14] Fan X., Chang L., Pu H., Zhao J., Wang H., Wang Y., He W., Huang J.R. (2024). Enhancement of Zein-Based Films for Mango Preservation Using High-Intensity Ultrasound and Castor Oil Plasticization. Ultrason. Sonochem..

[15] Nabgan M., Shariatifar N., Jahed-Khaniki G., Molaee-Aghaee E., Sani M.A. (2025). Development and Characterization of Whey Protein Packaging Film Loaded with Pickering Emulsion Stabilized Marjoram (*Origanum majorana* L.) Essential Oil. Int. J. Biol. Macromol..

[16] Gomes A., Cangussu L.B., Cunha R.L., de Oliveira L.S., Franca A.S., Costa A.L.R. (2025). Investigating the Impact of Chlorogenic Acid Content and Cellulose Nanoparticles on Sunflower Protein-Based Emulsions and Films. Foods.

[17] das Gracas Rodrigues Ferreira M., Costa C.J., Pinheiro C.U.B., Souza E.R.B., Carvalho C.O. (2022). *Mauritia flexuosa*—Buriti. Especies Nativas da Flora Brasileira de Valor Economico Atual ou Potencial: Plantas para o Futuro: Região Norte.

[18] Bispo A.G., Oliveira N.A., Cardoso C.X., Lima S.A.M., Job A.E., Osorio-Román I.O., Danna C.S., Pires A.M. (2018). Red-Light-Emitting Polymer Composite Based on PVDF Membranes and Europium Phosphor Using Buriti Oil as Plasticizer. Mater. Chem. Phys..

[19] Resende L.M., Franca A.S., Oliveira L.S. (2019). Buriti (*Mauritia flexuosa* L. f.) Fruit by-Products Flours: Evaluation as Source of Dietary Fibers and Natural Antioxidants. Food Chem..

[20] Durães J.A., Drummond A.L., Pimentel T.A.P.F., Murta M.M., Moreira S.G.C., Sales M.J.A. (2008). Thermal and Structural Behavior of Buriti Oil/Poly(Methyl Methacrylate) and Buriti Oil/Polystyrene Materials. J. Therm. Anal. Calorim..

[21] Anjos H.A., Castro D.A.M., dos Santos-Neto A.G., da Luz J.R.D., das Graças Almeida M., Leite Neta M.T.S., Narain N., Pagani A.A.C., Franceschi E., Hernández-Macedo M.L. (2023). Gelatin-Based Films Incorporated with Buriti Oil (*Mauritia flexuosa* L.) as Active Packaging for Artisanal Cheese Conservation. Bioresour. Technol. Rep..

[22] Freitas B.L.S., Almeida N.P., Haddad F.F., Oliveira L.S., Franca A.S. (2025). Evaluation of Buriti (*Mauritia flexuosa* L.) Oil as an Additive for Carbohydrate-Based Biodegradable Films. Foods.

[23] de Oliveira Alves L.T., Fronza P., Gonçalves I., da Silva W.A., Oliveira L.S., Franca A.S. (2024). Development of Polymeric Films Based on Sunflower Seed Proteins and Locust Bean Gum. Polymers.

[24] Siqueira R.A., Veras J.M.L., de Sousa T.L., de Farias P.M., de Oliveira Filho J.G., Bertolo M.R.V., Egea M.B., Plácido G.R. (2022). Pequi Mesocarp: A New Source of Pectin to Produce Biodegradable Film for Application as Food Packaging. Food Sci. Technol..

[25] Fronza P., Batista M.J.P.A., Franca A.S., Oliveira L.S. (2024). Bionanocomposite Based on Cassava Waste Starch, Locust Bean Galactomannan, and Cassava Waste Cellulose Nanofibers. Foods.

[26] Gontard N., Duchez C., Cuq J.-L., Guilbert S. (1994). Edible Composite Films of Wheat Gluten and Lipids: Water Vapour Permeability and Other Physical Properties. Int. J. Food Sci. Technol..

[27] Yan X., Wardana A.A., Wigati L.P., Meng F., Leonard S., Nkede F.N., Tanaka F., Tanaka F. (2023). Characterization and Bio-Functional Performance of Chitosan/Poly (Vinyl Alcohol)/Trans-Cinnamaldehyde Ternary Biopolymeric Films. Int. J. Biol. Macromol..

[28] Sarker A., Matak K., Jaczynski J. (2025). Effect of Transglutaminase Concentration on the Properties of Soy Protein Isolate-Pectin Composite Edible Films. Food Packag. Shelf Life.

[29] Salgado P.R., López-Caballero M.E., Gómez-Guillén M.C., Mauri A.N., Montero M.P. (2013). Sunflower Protein Films Incorporated with Clove Essential Oil Have Potential Application for the Preservation of Fish Patties. Food Hydrocoll..

[30] de Oliveira J.P., Silva F.N., Soares J.F., da Silva M.A.N., Felix P.H.C., de Oliveira Ferreira Rocha L., Ramos C.L., Sosa F.H.B., da Costa J.M.G. (2026). Structural Interactions and Technological and Sensory Effects of *Mauritia flexuosa* (Buriti) Oil Microparticles Microencapsulated with the Polysaccharides Gum Arabic and Inulin in the Yogurt Protein Matrix. Int. J. Biol. Macromol..

[31] Spinei M., Oroian M., Ursachi V.F. (2024). Characterization of Biodegradable Films Based on Carboxymethyl Cellulose and Citrus Pectin Films Enriched with Bee Bread Oil and Thyme Oil. LWT.

[32] Orliac O., Rouilly A., Silvestre F., Rigal L. (2003). Effects of Various Plasticizers on the Mechanical Properties, Water Resistance and Aging of Thermo-Moulded Films Made from Sunflower Proteins. Ind. Crops Prod..

[33] Rahman M.H., Bhoi P.R. (2021). An Overview of Non-Biodegradable Bioplastics. J. Clean. Prod..

[34] Wang X., Han Y., Wang H., Wang X., Li Y., Li S., Li H., Lei W., Liu X., Gao Z. (2026). Citrus Waste-Inspired Pectin Emulsion Films Activated with Essential Oils and Anthocyanins for Separate Antimicrobial and Indicator Applications. Carbohydr. Polym..

[35] Mahdy S.M., Selim S., Abu-Hussien S.H., Alahmadi A., Waznah M.S., Alzahofi N.N., Albalawi A.S., Albakri N.N., Almutrafy A.M., Salem S.S. (2025). Milk Protein-Based Edible Films Enriched with Clove and Camphor Essential Oils: Characterization, Bioactivity, and Potential Applications against Periodontal Pathogens. Int. J. Biol. Macromol..

[36] Chenah M., Karabulut G., Zannou O., Tosif M.M., Goksen G. (2026). Multi-Component Chitosan–Pectin Films Reinforced with Padina Pavonica Nanocellulose and Thymus Algeriensis Essential Oil for Red Meat Shelf-Life Enhancement. Food Chem. X.

[37] Huo J., Wang L., Su S., Yu X., Duan Y., Wang P., Xiao Z. (2025). Development and Characterization of Soy Protein Isolate-Based Emulsion Films with Green Sichuan Pepper Essential Oil: Functional and Structural Insights. Food Res. Int..

[38] He B., Gao Y., Lou W., Wang J., Xin S., Ma K., Li L. (2025). Biodegradable Food Packaging Films: Material Source, Mechanical and Water Vapor Barrier Properties, and Improvement Strategies—A Comprehensive Review. Food Res. Int..

[39] Yadav P., Mahapatra U., Sharma R., Rustagi S., Katyal M., Sharma M., Nayak P.K., Nath P.C., Sridhar K. (2025). Recent Advances in Plant Protein-Based Sustainable Edible Film and Coatings for Applications in the Food-Pharma Industry—Opportunities and Challenges: A Review. Int. J. Biol. Macromol..

[40] Becze A., Senila M., Senila L., Dordai L., Cadar O., Fuss-Babalau V.L., Roman M., Levei L., Uiuiu P., Naghiu M.O. (2025). Optimization of Protein Extraction from Sunflower Meal Using Taguchi Design and Regression Modeling for Human Nutrition Applications. Foods.

[41] Zhang W., Lu J., Zhao X., Xu X. (2022). An Optimized Approach to Recovering O/W Interfacial Myofibrillar Protein: Emphasizing on Interface-Induced Structural Changes. Food Hydrocoll..

[42] Lu H., Ma R., Chang R., Tian Y. (2021). Evaluation of Starch Retrogradation by Infrared Spectroscopy. Food Hydrocoll..

[43] Ursachi V.F., Oroian M., Spinei M. (2024). Development and Characterization of Biodegradable Films Based on Cellulose Derivatives and Citrus Pectin: A Comparative Study. Ind. Crops Prod..

[44] Liu X., Renard C.M.G.C., Bureau S., Le Bourvellec C. (2021). Revisiting the Contribution of ATR-FTIR Spectroscopy to Characterize Plant Cell Wall Polysaccharides. Carbohydr. Polym..

[45] da Costa D.S., Takeuchi K.P., da Silva R.M., de Oliveira Filho J.G., Bertolo M.R.V., Belisário C.M., Egea M.B., Plácido G.R. (2022). Cassava-Starch-Based Films Incorporated with Buriti (*Mauritia flexuosa* L.) Oil: A New Active and Bioactive Material for Food Packaging Applications. Polysaccharides.

[46] Souto R.N.M., da Silva Pinho J., Peixe C.L.D., Trindade M.E.F., de Souza P.G., da Silva P.E., Teixeira-Costa B.E., Castelo-Branco V.N., Teodoro A.J. (2025). Buriti (*Mauritia flexuosa* L.f.) and Acuri (*Attalea phalerata* Mart. Ex Spreng) Oils as Functional Lipid Sources in Bakery Products: Bioactive Composition, Sensory Evaluation, and Technological Performance. Foods.

[47] Wandee Y., Uttapap D., Mischnick P. (2019). Yield and Structural Composition of Pomelo Peel Pectins Extracted under Acidic and Alkaline Conditions. Food Hydrocoll..

[48] Han K., Liu Y., Liu Y., Huang X., Sheng L. (2020). Characterization and Film-Forming Mechanism of Egg White/Pullulan Blend Film. Food Chem..

[49] Qin C., Li Z., Zhang J., Meng H., Zhu C. (2024). Preparation, Physicochemical Properties, Antioxidant, and Antibacterial Activities of Quaternized Hawthorn Pectin Films Incorporated with Thyme Essential Oil. Food Packag. Shelf Life.

[50] Silva N.D.S.E., Hernández E.J.G.P., Araújo C.D.S., Joele M.R.S.P., de Fátima Henriques Lourenço L. (2018). Development and Optimization of Biodegradable Fish Gelatin Composite Film Added with Buriti Oil. CYTA-J. Food.

[51] Tirgarian B., Farmani J., Milani J.M. (2023). Edible Oleofilms with High Vegetable Oil Content Obtained from Novel Soy Protein Isolate/Gelatin/Chitosan Nanofiber Emulgels. Food Hydrocoll..

[52] Dilkushi H.A.S., Jayarathna S., Manipura A., Chamara H.K.B.S., Edirisinghe D., Vidanarachchi J.K., Priyashantha H. (2024). Development and Characterization of Biocomposite Films Using Banana Pseudostem, Cassava Starch and Poly(Vinyl Alcohol): A Sustainable Packaging Alternative. Carbohydr. Polym. Technol. Appl..

[53] Guo H., Xu Y., Chen H., Si X., Zhou M., Zhu E. (2024). Antagonistic Yeast and Trehalose-Enriched Gelatin Film: A Bioactive Antifungal Packaging Film for Cherry Tomato Preservation. Food Packag. Shelf Life.

[54] Bhatia S., Al-Harrasi A., Shah Y.A., Alrasbi A.N.S., Jawad M., Koca E., Aydemir L.Y., Alamoudi J.A., Almoshari Y., Mohan S. (2024). Structural, Mechanical, Barrier and Antioxidant Properties of Pectin and Xanthan Gum Edible Films Loaded with Grapefruit Essential Oil. Heliyon.

[55] Nilsson F., Elf P., Capezza A., Wei X., Tsegaye B., Polisetti V., Svagan A.J., Hedenqvist M. (2025). Environmental Concerns on Water-Soluble and Biodegradable Plastics and Their Applications—A Review. Sci. Total Environ..

[56] Hadidi M., Jafarzadeh S., Forough M., Garavand F., Alizadeh S., Salehabadi A., Khaneghah A.M., Jafari S.M. (2022). Plant Protein-Based Food Packaging Films; Recent Advances in Fabrication, Characterization, and Applications. Trends Food Sci. Technol..

[57] Cai Z., Li M., Zhu Z., Wang X., Huang Y., Li T., Gong H., Yan M. (2023). Biological Degradation of Plastics and Microplastics: A Recent Perspective on Associated Mechanisms and Influencing Factors. Microorganisms.

[58] Tokiwa Y., Calabia B.P., Ugwu C.U., Aiba S. (2009). Biodegradability of Plastics. Int. J. Mol. Sci..

[59] Mohammadi M., Fasihi M. (2025). Eco-Friendly Polylactic Acid/Modified Thermoplastic Starch Films Enhanced with Clove Essential Oil and Cochineal for Dual-Functional Active and Intelligent Food Packaging. Carbohydr. Polym..

[60] Patil S., Jalgaonkar K., Bharimalla A.K., Mahawar M.K., Dhakane-Lad J., Raja A.S.M. (2025). PBAT-Based Nanocomposite Blown Films: A Green Alternative for Packaging of Peeled Garlic. Polymer.

