# Supplementary material for: Impact of Buriti Oil Incorporation on the Structural and Functional Properties of Polymeric Films Based on Pectin and Sunflower Seed Protein
Source: Foods. 2026 Oct 22;15(21):3779. doi: 10.3390/foods15213779 (PMC13763140; doi:10.3390/foods15213779)
Supplement: Supplementary file 1 [file foods-15-03779-s001.zip › foods-4585923-supplementary.pdf]

Supplementary Materials

Table S1. Statistical parameters and goodness-of-fit for the mathematical deconvolution of the Amide I region (1600–1700 cm^{-1}) in the FTIR spectra of the films.

Polymer	Film	BaseLine	X^2	R$^2_{adj}$	RSS	df	*n*
SSP	F1	Spline	2.0024E-07	9.9931E-01	1.7220E-05	86	107
	F2	Spline	3.9620E-08	9.9920E-01	3.4073E-06	86	107
	F3	Spline	1.7669E-08	9.9904E-01	1.5196E-06	86	107
Pectin/SSP (1:3)	F1	Spline	7.9236E-08	9.9938E-01	6.8143E-06	86	107
	F2	Spline	1.4883E-08	9.9788E-01	1.3246E-06	89	107
	F3	Spline	6.3885E-09	9.9922E-01	5.4941E-07	86	107

(F1) control films; (F2) films with partial replacement of glycerol with buriti oil emulsion; (F3) films with total replacement of glycerol with buriti oil emulsion. χ^2: Chi-square; R$^2_{adj}$: Adjusted R-square; RSS: Residual Sum of Squares; df: Degrees of freedom; *n*: Number of data points

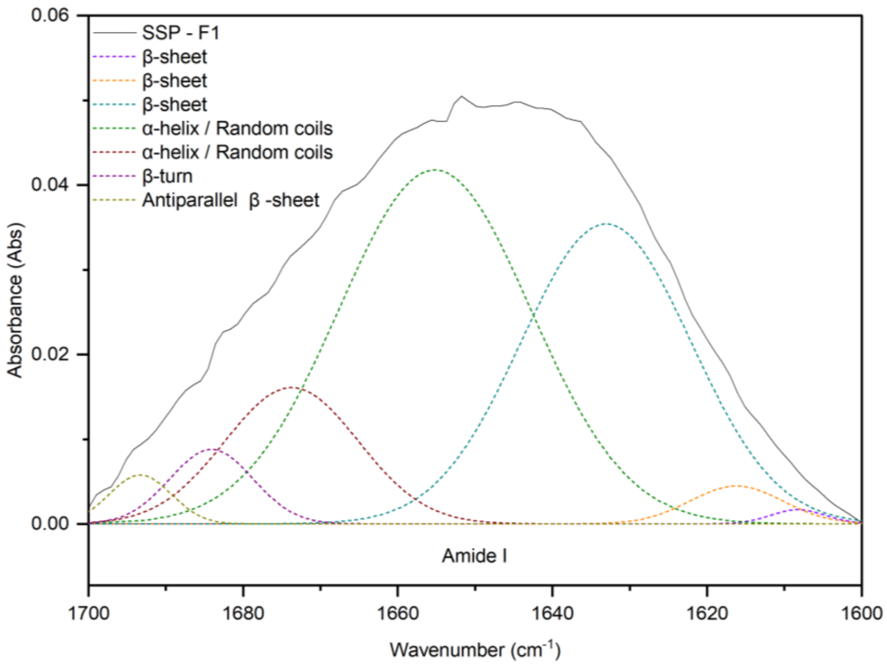

Figure S1. FTIR amide I peak deconvolution of SSP - F1

Table S2. Peak assignments and relative areas of secondary structures obtained from the mathematical deconvolution of the Amide I region (1600–1700 cm^{-1}) for the SSP-F1 film.

Polymer	Film	Peak	Peak Type	Wavenumber (cm^{-1})	Secondary structure	Relative Area (%)	Total Area (%)
SSP	F1	1	Gaussian	1617	intra- or intermolecular β-sheet	0.5185	36.7101
		2	Gaussian	1624	intra- or intermolecular β-sheet	2.3328	
		3	Gaussian	1636	intramolecular β-sheet	33.8588	
		4	Gaussian	1651	α-helix / Random coils	45.0879	57.1810
		5	Gaussian	1667	α-helix / Random coils	12.0931	
		6	Gaussian	1682	β-turn	4.1176	4,1176
		7	Gaussian	1694	antiparallel β -sheet	1.9914	1,9914

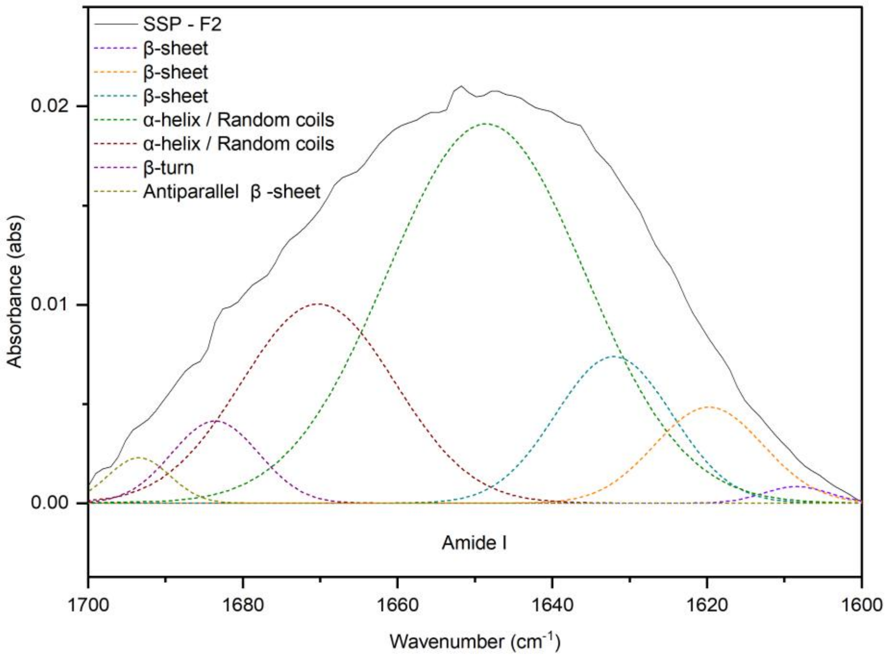

Figure S2. FTIR amide I peak deconvolution of SSP - F2.

Table S3. Peak assignments and relative areas of secondary structures obtained from the mathematical deconvolution of the Amide I region (1600–1700 cm^{-1}) for the SSP-F2 film.

Polymer	Film	Peak	Peak Type	Wavenumber (cm^{-1})	Secondary structure	Relative Area (%)	Total Area (%)
SSP	F2	1	Gaussian	1617	intra- or intermolecular β-sheet	0.7485	19.8314
		2	Gaussian	1624	intra- or intermolecular β-sheet	7.2232	
		3	Gaussian	1636	intramolecular β-sheet	11.8596	
		4	Gaussian	1651	α-helix / Random coils	52.0574	73.2755
		5	Gaussian	1668	α-helix / Random coils	21.2181	
		6	Gaussian	1682	β-turn	4.9704	4.9704
		7	Gaussian	1694	antiparallel β -sheet	1.9228	1.9228

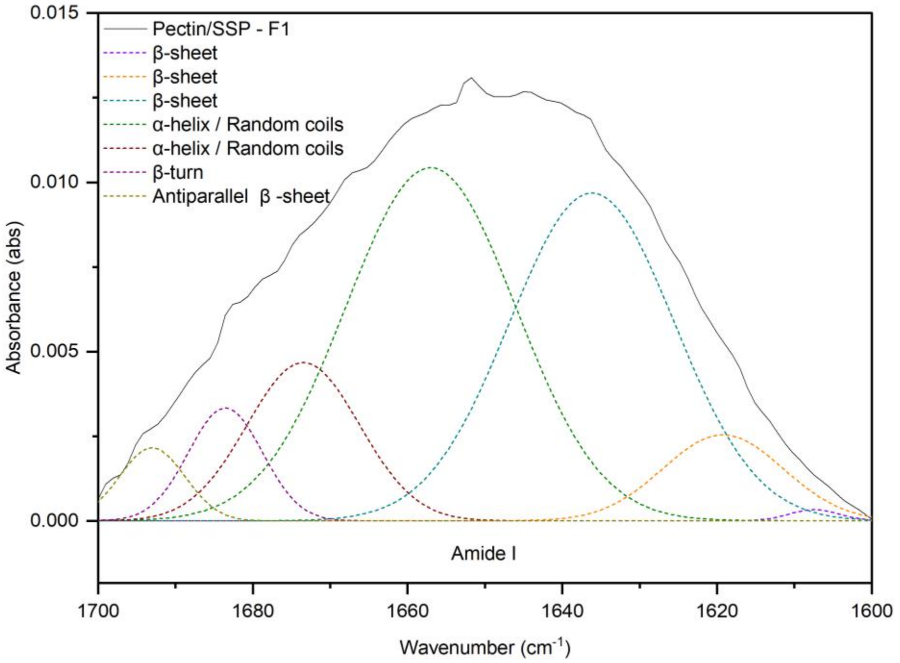

Figure S3. FTIR amide I peak deconvolution of SSP - F3.

Table S4. Peak assignments and relative areas of secondary structures obtained from the mathematical deconvolution of the Amide I region (1600–1700 cm^{-1}) for the SSP-F3 film.

Polymer	Film	Peak	Peak Type	Wavenumber (cm^{-1})	Secondary structure	Relative Area (%)	Total Area (%)
SSP	F3	**1**	Gaussian	1617	intra- or intermolecular β-sheet	0.3629	41.6050
		**2**	Gaussian	1624	intra- or intermolecular β-sheet	6.6244	
		**3**	Gaussian	1636	intramolecular β-sheet	34.6177	
		**4**	Gaussian	1651	α-helix / Random coils	38.5294	49.9807
		**5**	Gaussian	1668	α-helix / Random coils	11.4513	
		**6**	Gaussian	1682	β-turn	5.3815	5.3815
		**7**	Gaussian	1694	antiparallel β -sheet	3.0329	3.0329

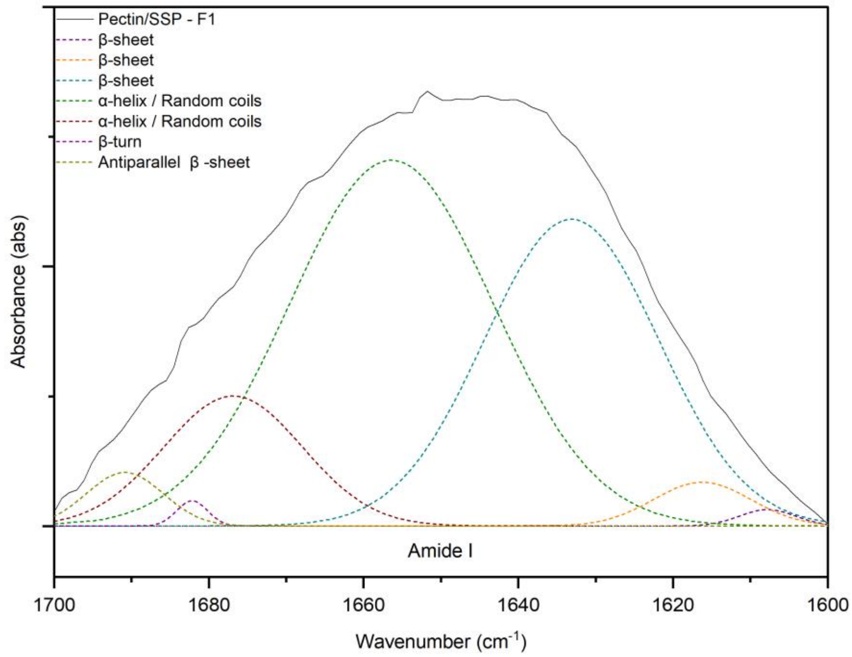

Figure S4. FTIR amide I peak deconvolution of Pectin/SSP - F1.

Table S5. Peak assignments and relative areas of secondary structures obtained from the mathematical deconvolution of the Amide I region (1600–1700 cm^{-1}) for the Pectin/SSP-F1 film.

Polymer	Film	Peak	Peak Type	Wavenumber (cm^{-1})	Secondary structure	Relative Area (%)	Total Area (%)
Pectin/SSP (1:3)	F1	1	Gaussian	1617	intra- or intermolecular β-sheet	0.5831	37.5475
		2	Gaussian	1624	intra- or intermolecular β-sheet	2.7195	
		3	Gaussian	1636	intramolecular β-sheet	34.2449	
		4	Gaussian	1651	α-helix / Random coils	47.8092	59.3482
		5	Gaussian	1667	α-helix / Random coils	11.5390	
		6	Gaussian	1682	β-turn	0.5007	0.5007
		7	Gaussian	1694	antiparallel β -sheet	2.6036	2.6036

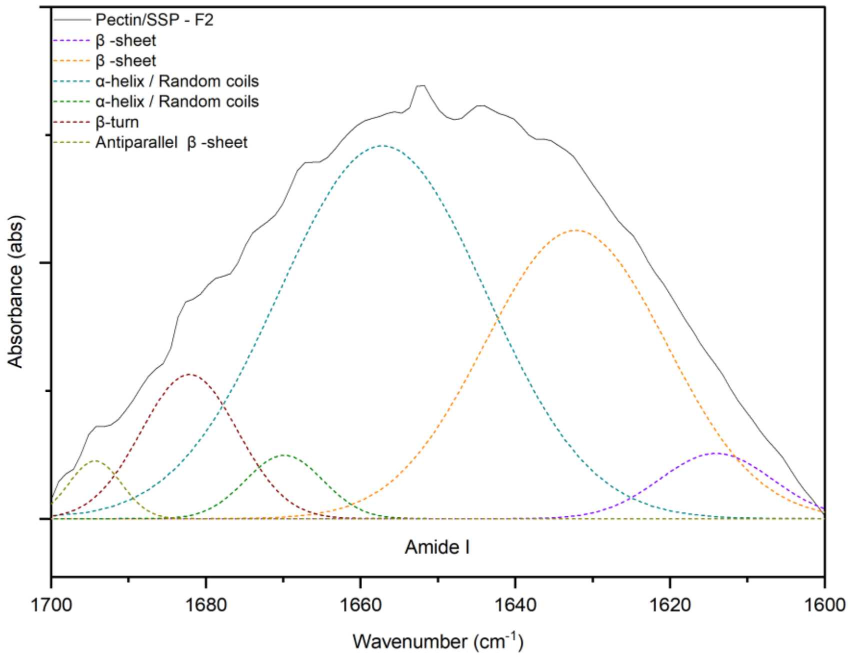

Figure S5. FTIR amide I peak deconvolution of Pectin/SSP - F2.

Table S6. Peak assignments and relative areas of secondary structures obtained from the mathematical deconvolution of the Amide I region (1600–1700 cm^{-1}) for the Pectin/SSP-F2 film.

Polymer	Film	Peak	Peak Type	Wavenumber (cm^{-1})	Secondary structure	Relative Area (%)	Total Area (%)
Pectin/SSP (1:3)	F2	**1**	Gaussian	1624	intra- or intermolecular β-sheet	4.5200	37.5225
		**2**	Gaussian	1635	intramolecular β-sheet	33.0025	
		**3**	Gaussian	1651	α-helix / Random coils	48.6624	51.7675
		**4**	Gaussian	1668	α-helix / Random coils	3.1051	
		**5**	Gaussian	1682	β-turn	8.8025	8.8025
		**6**	Gaussian	1694	antiparallel β -sheet	1.9075	1.9075

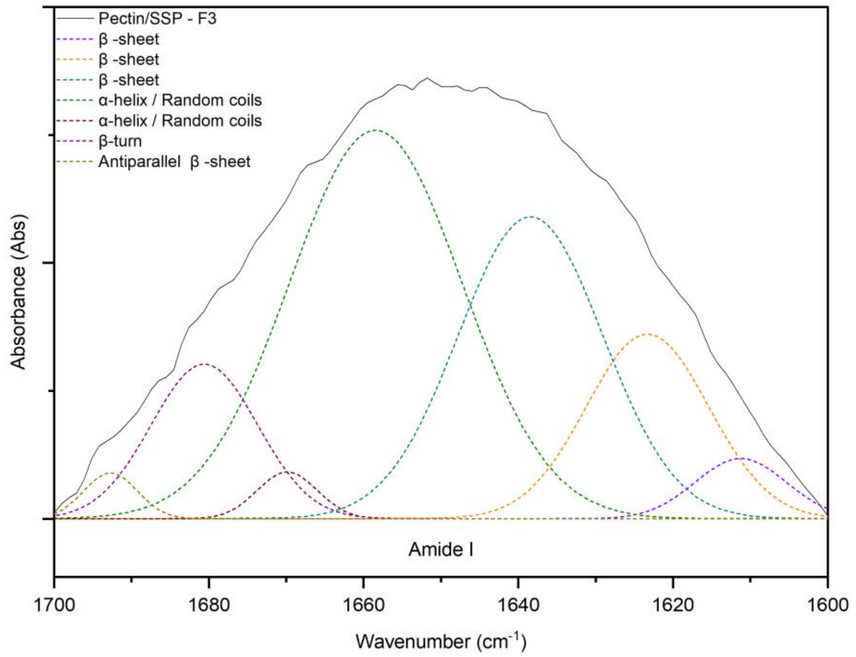

Figure S6. FTIR amide I peak deconvolution of Pectin/SSP - F3.

Table S7. Peak assignments and relative areas of secondary structures obtained from the mathematical deconvolution of the Amide I region (1600–1700 cm^{-1}) for the Pectin/SSP-F3 film.

Polymer	Film	Peak	Peak Type	Wavenumber (cm^{-1})	Secondary structure	Relative Area (%)	Total Area (%)
Pectin/SSP (1:3)	F3	**1**	Gaussian	1617	Intra- or intermolecular β-sheet	3.3421	44.6504
		**2**	Gaussian	1624	intra- or intermolecular β-sheet	14.0807	
		**3**	Gaussian	1636	intramolecular β-sheet	27.2276	
		**4**	Gaussian	1651	α-helix / Random coils	41.8064	43.5621
		**5**	Gaussian	1668	α-helix / Random coils	1.7557	
		**6**	Gaussian	1682	β-turn	10.2623	10.2623
		**7**	Gaussian	1694	antiparallel β -sheet	1.5253	1.5253
